# Two 3′-*O*-β-glucosylated nucleoside fluorometabolites related to nucleocidin in *Streptomyces calvus*†
†Electronic supplementary information (ESI) available. See DOI: 10.1039/c9sc03374b


**DOI:** 10.1039/c9sc03374b

**Published:** 2019-08-20

**Authors:** Xuan Feng, Davide Bello, Phillip T. Lowe, Joshua Clark, David O'Hagan

**Affiliations:** a School of Chemistry , University of St Andrews , North Haugh, St Andrews , Fife , KY16 9ST , UK . Email: do1@st-andrews.ac.uk

## Abstract

The antibiotic nucleocidin is a product of the soil bacterium *Streptomyces calvus* T-3018. It is among the very rare fluorine containing natural products but is distinct from the other fluorometabolites in that it is not biosynthesised from 5′-fluorodeoxyadenosine *via* the fluorinase. It seems to have a unique enzymatic fluorination process. We disclose here the structures of two 4′-fluoro-3′-*O*-β-glucosylated metabolites (F-Mets I and II) which appear and then disappear before nucleocidin production in batch cultures of *S. calvus*. Full genome sequencing of *S. calvus* T-3018 and an analysis of the putative biosynthetic gene cluster for nucleocidin identified UDP-glucose dependent glucosyl transferase (*nucGT*) and glucosidase (n*ucGS*) genes within the cluster. We demonstrate that these genes express enzymes that have the capacity to attach and remove glucose from the 3′-*O*-position of adenosine analogues. In the case of F-Met II, deglucosylation with the NucGS glucosidase generates nucleocidin suggesting a role in its biosynthesis. Gene knockouts of *nucGT* abolished nucelocidin production.

## Abstract 2

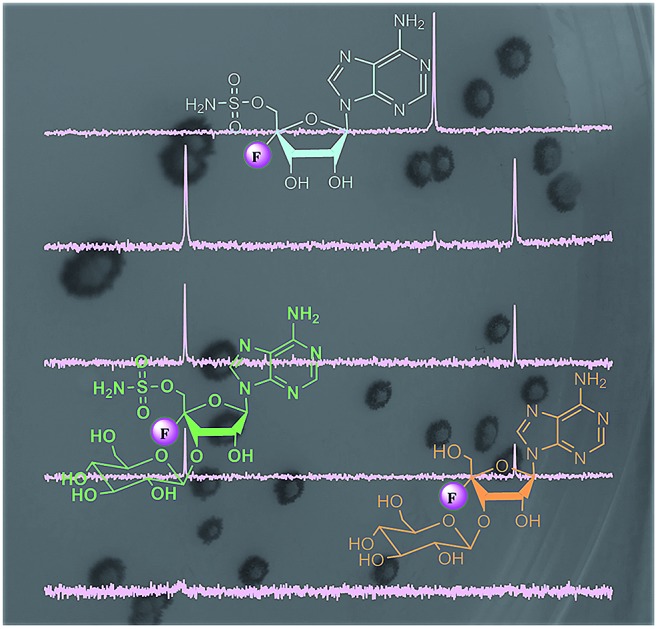
Two novel 3′-*O*-β-glucosylated nucleosides are identified from *Streptomyces calvus* fermentations prior to nucleocidin production, suggesting an early role during the biosynthesis of this antibiotic.

## Introduction

**Fig. 1:**
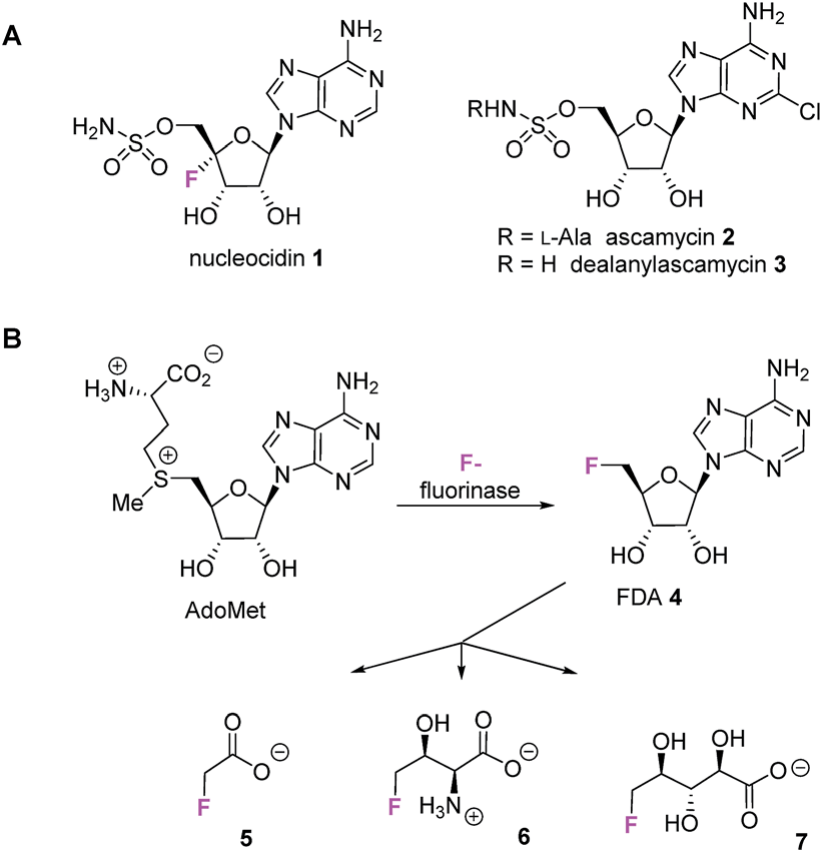
(A) Nucleoside antibiotics **1–3**. (B) The fluorinated natural products **5–7** derive from the fluorinase *via* FDA **4**, however this enzyme is not involved in nucleocidin **1** biosynthesis.

The antibiotic nucleocidin **1** is among the very rare fluorinated natural products.^1^ It is a 5′-*O*-sulfonyl antibiotic produced by the actinomycete bacterium *Streptomyces calvus.*^2^ Nucleocidin **1** is a member of a small class of such antibiotics which include the chlorine containing ascamycin **2** and dealanylascamycin **3**, as shown in Fig. 1A.^3^

The fluorine atom unique to nucleocidin **1** is located at C4′ of the ribose ring of the 5′-*O*-sulfamylated adenosine. The biosynthesis of nucleocidin is unknown. Only three other bacterial fluorometabolites have been identified: these are fluoroacetate **5**,^4^,^5^ 4-fluorothreonine **6** 4,5 and (2*R*,3*S*,4*S*)-trihydroxy-5-fluoropentanoic acid **7**,^6^ however these metabolites are biosynthesised by pathways which are initiated by the C–F bond forming enzyme, adenosyl fluoride synthase (EC 2.5.1.63) also known as ‘fluorinase’.^7^ This enzyme combines fluoride ion and *S*-adenosyl-l-methionine to generate 5′-fluorodeoxy adenosine **4** (FDA) which is then biotransformed to the bacterial fluorometabolites **5–7** as summarised in Fig. 1B.^1^,^8^ However, full genome sequencing of the nucleocidin **1** producer *S. calvus*^9^ indicates that there is no related fluorinase gene present in *S. calvus* and the positioning of the fluorine at the C-4′ position of the ribose ring in nucleocidin **1** is inconsistent with an origin from FDA **4**, suggesting a unique fluorination enzyme.

For many years nucleocidin **1** production remained elusive because the publically available strains of *S. calvus* did not support its biosynthesis. Recently however, this was recognised to be the result of a bald gene (*bld A*) mutation which when reversed in *S. calvus* ATCC 13382, re-established nucleocidin production as well as an aerial mycelial phenotype.^9^,^10^ In this study we have used a producing strain of *S. calvus* T-3018 held by Pfizer, which does not have this mutation and is a competent nucleocidin **1** producer. In our efforts to explore the biosynthesis of nucleocidin **1** we recently reported the incorporation of isotopically labelled glycerols into **1** and demonstrated that both of the C5′-hydrogens of the ribose ring derive intact from the *pro-R* hydroxymethyl group of glycerol. This indicates that there is no oxidation at this carbon as glycerol is progressed along the pentose phosphate pathway and into the ribose moiety of nucleocidin.^11^ This places certain limitations on the biosynthesis of **1** by excluding late stage intermediates that might activate C4′ for fluorination, by oxidation at C5′.

When monitoring *S. calvus* T-3018 cultures by ^19^F{^1^H}-NMR to assess nucleocidin **1** production, periodically two unidentified fluorinated metabobiles (F-Mets I and II) appear.^11^ These compounds have also been observed in other studies on nucleocidin **1** production in *S. calvus* ATCC 13382 9*a* and most recently during nucleocidin production in *Streptomyces asteroporus* DSM 41452.^10^

**Fig. 2:**
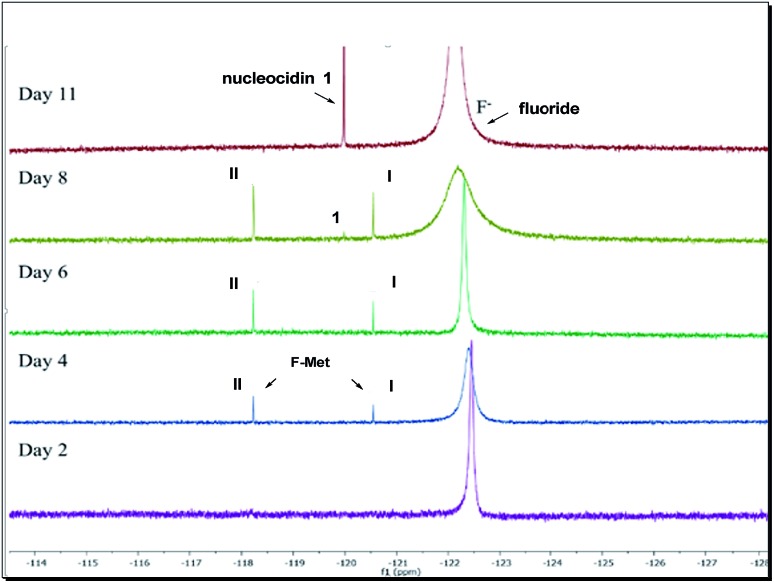
^19^F-NMR time course of nucleocidin **1** production in *S. calvus* showing the appearance and disappearance of F-Mets I and II over 11 days.

Our observations indicate that F-Mets I and II appear and disappear prior to the accumulation of nucleocidin **1** itself, suggesting an intimate association with antibiotic production. A typical time course profile from a *S. calvus* T-3018 fermentation is shown in Fig. 2 and it can be seen that F-Mets I and II accumulate between days 4 and 8 and then disappear between days 8–11 as nucleocidin **1** emerges. Clearly the structure of these compounds could offer an insight into the fluorometabolite pathway in *S. calvus*. We are now able to report their structures and have identified genes/enzymes which lead to their formation and in one case its conversion to nucleocidin **1**.

## Results and discussion

F-Mets I and II were isolated in low milligram amounts by preparative HPLC guided by MS–MS analysis after adsorption onto charcoal from 6–8 day cultures of *S. calvus* T-3018. High resolution mass spectrometry (HRMS) gave accurate masses and therefore elemental constitutions (F-Met I = 448.14673 amu; C_16_H_23_FN_5_O_9_. F-Met II = 527.11993 amu; C_16_H_24_FN_6_O_11_S). Although the titres were low, sufficient material was isolated to be able to record ^1^H- and ^19^F-NMR in each case and a ^13^C-NMR for F-Met II. Detailed 2D-NMR (COSY, TOCSY and HMBC) analyses indicated that the compounds were the 3′-*O*-β-glucosylated derivatives of **1** and that F-Met I is a 3′-*O*-β-glucosylated-4′-fluoroadenosine and F-Met II is a 3′-*O*-β-glucosylated nucleocidin. The NMR data are summarised in Table 1 alongside nucleocidin **1** for comparison. See ESI† for full details. The structure of F-Met II is illustrated in a book chapter from 1995, indicating a compound isolated from a Ciba-Geigy bacterium, *Streptomyces* strain R-156, however we cannot find any details of its isolation or characterisation beyond this anecdotal comment.^12^

**Table 1:** ^1^H and ^13^C NMR data of F-Mets I, II and nucleocidin **1** (700 MHz, acetone-d_6_)

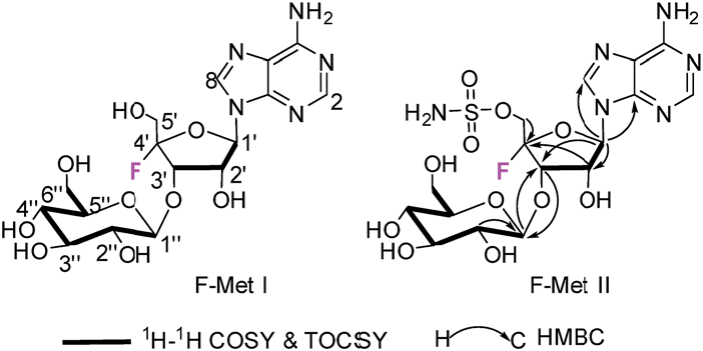					
Position	F-Met I (*δ*_F_ = –120.54 ppm)	F-Met II (*δ*_F_ = –118.23 ppm)		Nucleocidin 1 (ref. 9) (*δ*_F_ = –119.98 ppm)	
	*δ*_H_ ppm	*δ*_H_ ppm	*δ*_C_ ppm	*δ*_H_ ppm	*δ*_C_ ppm
2	8.22 (s)	8.36 (s)	—	8.21(s)	152.9
8	8.29 (s)	8.38 (s)	139.2	8.21(s)	139.4
1′	6.37 (d, 2.0)	6.45 (d, 0.9)	90.9	6.39 (d, 1.9)	91.2
2′	4.83 (dd, 5.6, 2.0)	4.91 (dd, 5.9, 1.4)	72.6	4.85 (dd, 6.5, 1.9)	71.9
3′	5.16 (dd, 16.7, 5.9)	5.35 (dd, 18.3, 6.0)	76.8	5.10 (m)	70.7
4′	—	—	114.7	—	115.9
5′ H^a^	3.76 (dd, 11.8, 4.8)	4.42 (d, 9.1),	66.5	4.35, 4.39 (m)	66.9
5′ H^b^	3.85 (overlap with 6′′ H^b^)	4.46 (d, 8.3)			
1′′	4.70 (d, 7.7)	4.76 (d, 7.7)	102.9	—	—
2′′–5′′	3.39–3.47 (m)	3.41–3.48 (m)	69.9–77.0	—	—
6′′ H^a^	3.69 (dd, 11.8, 4.9)	3.69 (m)	61.3	—	—
6′′ H^b^	3.84 (overlap with 5′ H^b^)	3.86 (m)			

**Fig. 3:**
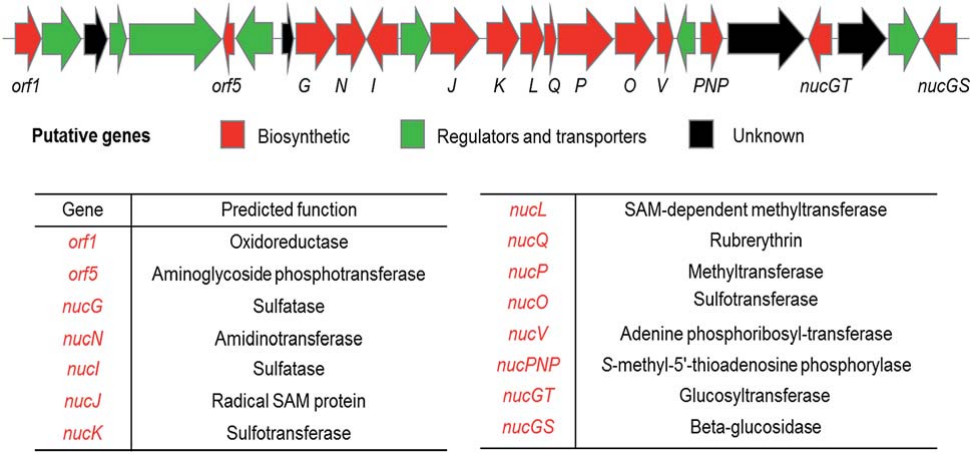
Organisation of the putative nucleocidin biosynthetic gene cluster in *S. calvus*. A fuller annotation and a comparison with the ascamycin **2** antibiotic gene cluster, can be found in the ESI Fig. S36.†

A putative gene cluster was previously identified by Zechel and Bechthold^9^ for nucleocidin **1** biosynthesis in *S. calvus* ATCC 13382 by homology to the gene cluster of the structurally related antibiotics ascamycins **2** and **3**.^13^ A summary of the gene cluster is illustrated in Fig. 3 and more comprehensively in Fig. S36.†

The integrity of the gene cluster was established when correcting the mutation in a *bld* gene, which codes a Leu-*t*RNA^UUA^ able to translate a rare TTA codon into leucine. Three genes in the *nuc* cluster (*nucJ*, *orf4* and *orf6*) contain this rare TTA codon and when the mutation in the *bldA* gene was corrected to re-establish this Leu-*t*RNA^UUA^ the production of nucleocidin was re-established. We have independently sequenced the *S. calvus* T-3018 genome (recently deposited in GenBank)^14^ and find that unlike *S. calvus* ATCC 13382, *S. calvus* T-3018 does not contain a *bld* gene mutation.

The genome of *S. asterosporus* DSM 41452 was recently sequenced^10^ and was shown to possess a similar cluster; when this organism was grown with fluoride it produced nucleocidin **1**, adding further support that this biosynthetic gene cluster is responsible for nucleocidin biosynthesis.

There is also a 3′-phosphoadenosine 5′-phosphosulfate synthases (PAPS) cassette within the genomes of these organisms, consistent with a requirement to assemble the sulfamoyl moiety of the antibiotic. From the *S. calvus* sequences, the putative *nuc* cluster predicts glycosyltransferase (*nucGT*) and β-glucosidase (*nucGS*) genes which sit at the end of the cluster and are located three genes apart (see Fig. S36† for details). In the light of the structures of F-Mets I and II, we were motivated to over-express these two candidate glucosylating genes to assay their products. This was achieved by PCR amplification from genomic DNA (*S. calvus*), insertion of the gene into a pEHISTEV plasmid and then over- expression in *E. coli* Rosetta cells.^5^,^15^ In each case soluble protein of the predicted molecular mass (35.5 kDa for NucGT and 53.6 kDa for NucGS) was obtained after purification on a nickel column (see ESI†). The proteins were unambiguously confirmed by MS–MS analyses. The over-expressed glycosyltransferase (NucGT), was explored as a catalyst for 3′-*O*-β-glucosylation of adenosine **8** and sulfamoyladenosine **9**^16^ as candidate substrates using the common glucose donor, UDP-glucose.^17^

**Fig. 4:**
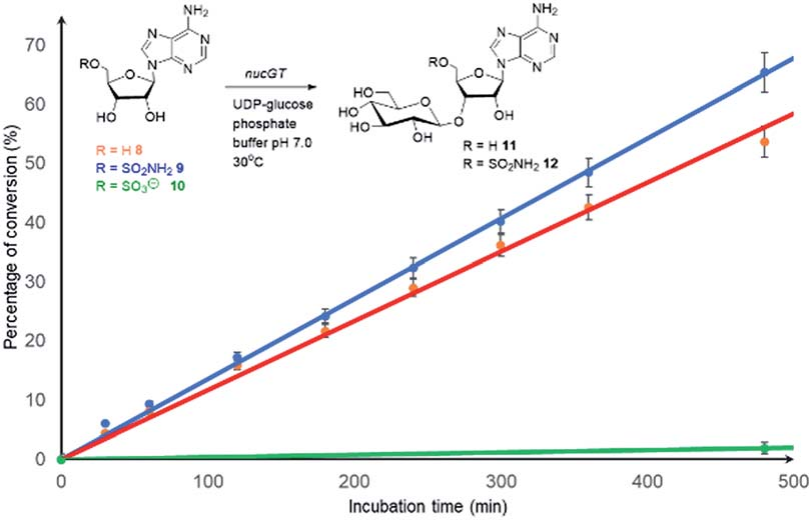
Comparison of the initial rates (triplicates) of **8–10** with Nuc-GT. Rates **8** (
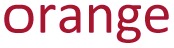
), **9** (
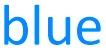
) and **10** (
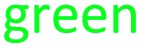
).

**Fig. 5:**
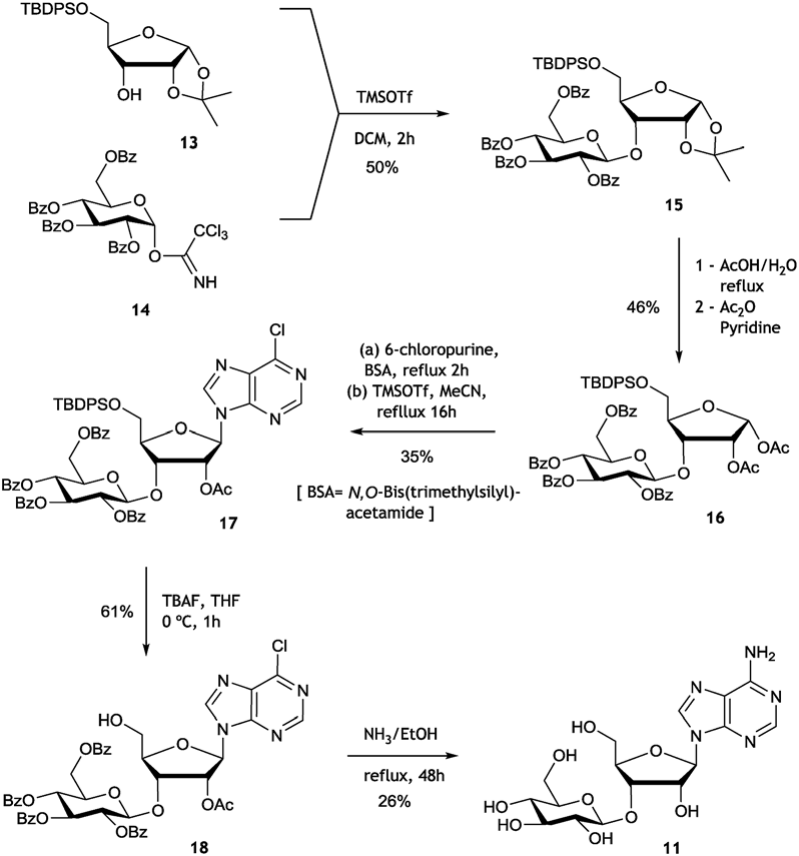
Synthesis route to 3′-*O*-β-*gluco*-adenosine **11**.

Both **8** and **9** were good substrates based on initial rates with a saturating concentration of UDP-glucose to generate **11** and **12** respectively (Fig. 4). Sulfamoyl adenosine **9** was the more efficient substrate. Adenosine-5′-sulfate **10**^18^ was also examined as a substrate for NucGT however it was not processed by the enzyme, and presumably the formal charge of the sulfate is incompatible with enzyme binding. Additional support for the product structures was secured by an independent synthesis of 3′-*O*-β-*gluco*-adenosine **11** as illustrated in Fig. 5. The synthesis involved a β-glycoside specific coupling of protected ribose **13**^19^ and activated glucose **14**^20^ to generate **15**. Introduction of the adenosine was then achieved *via* a 6-chloropurine strategy^21^ to generate **18**, with global aminolysis completing the synthesis. Synthetic and enzymatically generated **11** were identical (see Fig. S34 and S35†). This synthesis also offers reinforcement of the structures of F-Mets I and II in terms of NMR signatures consistent with 3′-*O*-glucosylation and β-glycoside linkage.

**Fig. 6:**
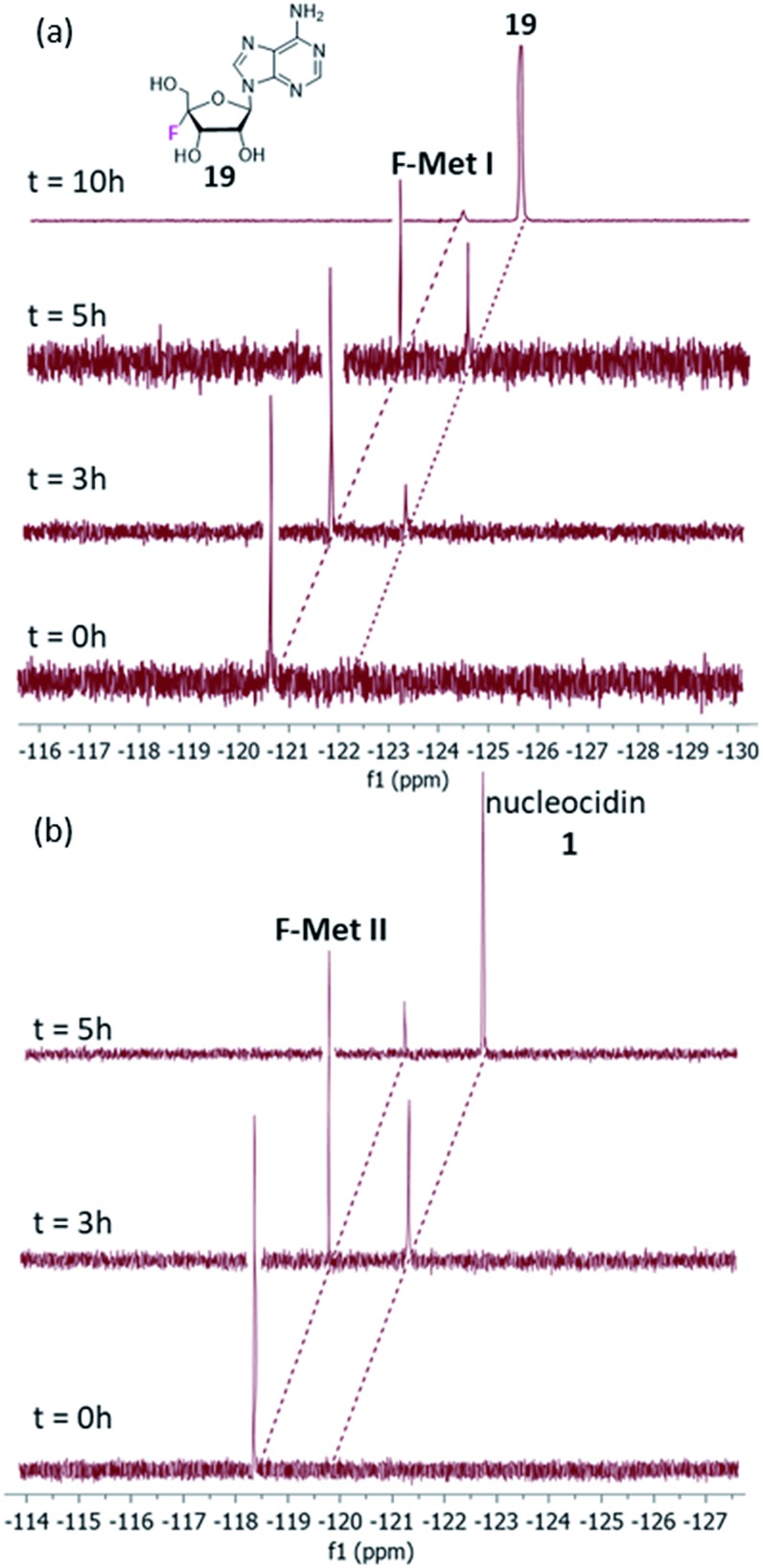
^19^F{^1^H}-NMR time course profiles of biotransformations of F-Mets I and II with β-glucosidase NucGS. (a) F-Met I is converted to 4′-fluoroadenosine **19**. (b) F-Met II is converted to nucleocidin **1**.

In order to assay the glucosidase (Nuc-GS) in the context of nucelocidin biosynthesis we required access to samples of F-Mets I and II. These are not readily synthesised, and thus analytical samples of each were secured by semi-preparative HPLC from *S. calvus* T-3018 fermentations arrested at day 8. Although only analytical amounts of material were isolated in each case, reactions could be conveniently monitored by ^19^F{^1^H}-NMR. Incubation of F-Met I with Nuc-GS resulted in its complete conversion to 4′-fluoroadenosine **19** as illustrated in Fig. 6a. This compound has previously been prepared and is reported to be stable in buffer for several days but it slowly hydrolyses to release fluoride.^22^

Incubation of F-Met I with NucGS gave a clear conversion to a new product with a signal at –122.5 ppm by ^19^F-NMR as illustrated in Fig. 6a, consistent with literature values for **19** and LC-MS data which also indicated the formation of **19**. On the other hand, treatment of F-Met II with NucGS resulted in its conversion to nucleocidin **1**, as illustrated in Fig. 6b. These *in vitro* biotransformations are consistent with the observed time course experiments illustrated in Fig. 2 and can be interpreted that F-Met I is deglucosylated to **19** and slowly decomposes to fluoride. If compound **19** survives the extraction after adsorption onto charcoal/Celite from the whole cells of *S. calvus*, then its presence would be disguised in the ^19^F NMR spectrum by the dominant fluoride ion peak at –122.5 ppm (see Fig. 2). For F-Met II, the action of NucGS generates nucleocidin **1** and it follows that NucGS and F-Met II could be involved in the final step in nucleocidin biosynthesis.

In order to further interrogate the roles of NucGS and NucGT in nucleocidin biosynthesis, knockout experiments were conducted for *nucGT* and *nucGS* (see Fig. S37 and S38†). In-frame deletion of *nucGT* completely abolished fluorometabolite production, consistent with a role in nucleocidin biosynthesis. A similar deletion of *nucGS* did not significantly affect the production of F-Mets I and II, consistent with their glucosylated status, however nucleocidin **1** production was not affected either. On further analysis of the *S. calvus* genome there are two additional β-glycosidase enzymes coded for elsewhere on the chromosome which have high (∼88%) amino acid sequence similarity to NucGS (see Fig. S39†) suggesting redundancy in terms of these enzymes with their capacity to deglucosylate F-Met II. We note that in the recently reported genome sequence of the nucleocidin producer, *Streptomyces asteroporus* DSM 41452 10 there are corresponding genes with a 100% amino acid similarity. Thus from the two organisms known to produce nucelocidin, both have these genes in their clusters, indicative of key roles in the biosynthesis.

**Fig. 7:**
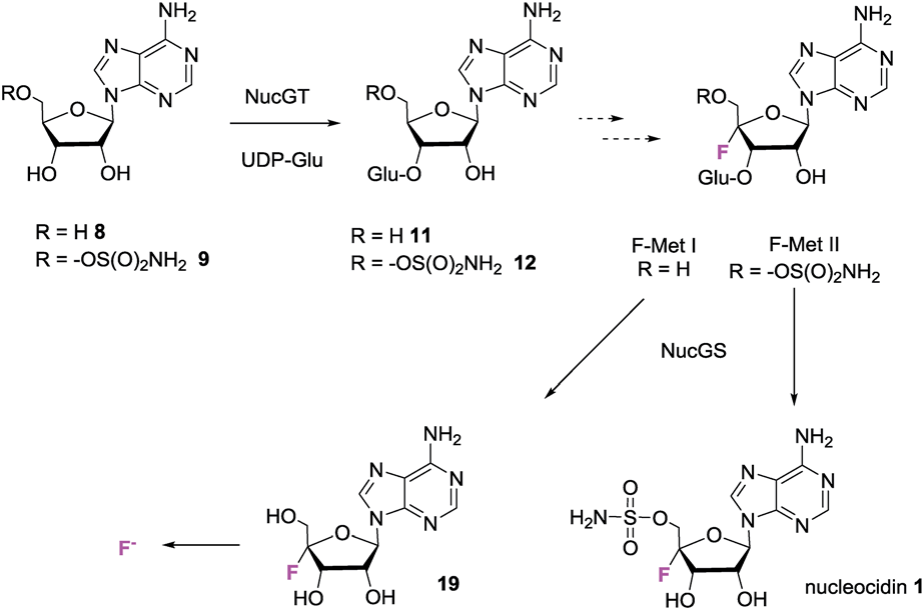
Putative minimal pathway for nucleocidin **1** biosynthesis.

The previous isotopic labelling studies with glycerol^11^ indicate that C5′ of adenosine is not oxidised during the biosynthesis of **1**, and this study suggests that the fluorination substrate is a 3′-*O*-β-glycosylated adenosine as summarised in Fig. 7.

It follows that an enzymatic fluorination mechanism involving oxidation of free alcohols at C3′ and/or C5′ seem unlikely, raising the prospect that fluorination occurs by direct C4′–H activation. One hypothesis would involve a C4′-centred oxocarbenium ion which is attacked by fluoride ion. Within the biosynthetic gene cluster are a number of genes encoding enzymes of unknown function and also a gene (*nucJ*) predicted to encode a radical SAM/iron–sulfur (Fe–S) cluster enzyme which may have the capacity to achieve such a two electron oxidation.

## Conclusions

We have identified two novel fluorometabolites (F-Met I and F-Met II), which are 3′-*O*-glucosylated, 4′-fluoro-riboadenosines from *S. calvus*. The products of two genes (*nucGS* and *nucGT*) identified in the putative nucleocidin gene cluster were over-expressed and the corresponding proteins have the ability to glucosylate (NucGT) and deglucosylate (NucGS) these two fluorometabolites at the 3′-hydroxyl group of the ribose ring. In the case of F-Met II, deglucosyaltion generates nucleocidin **1**. These observations support a role for F-Met I and F-Met II in the biosynthesis of nucleocidin. The identity of these metabolites is highly suggestive that one is a product of the unknown fluorination enzyme in *S. calvus*, and should provide a greater focus In efforts to identify this interesting enzyme.

## Conflicts of interest

The authors declare no conflicts of interest.

## Supplementary Material

Supplementary information
