# Supplementary material for: Two 3′-O-β-glucosylated nucleoside fluorometabolites related to nucleocidin in Streptomyces calvus
Source: Chem Sci. 2019 Aug 20;10(41):9501–5. doi: 10.1039/c9sc03374b (PMC7017864; doi:10.1039/c9sc03374b)
Supplement: Supplementary file 1 [file SC-010-C9SC03374B-s001.pdf]

Electronic Supplementary Material (ESI) for Chemical Science.
This journal is © The Royal Society of Chemistry 2019

General experimental procedures

NMR spectra were recorded at 298 K on Bruker Advance II 400, Advance III HD 500 or Advance III HD 700 instruments. ^{1}H and ^{13}C NMR spectra were recorded in deuterated solvent as the lock and the residual solvent as the internal standard. ^{19}F NMR spectra were recorded using $CFCl_3$ as an external reference. Chemical shifts are reported in parts per million (ppm) and coupling constants (*J*) are reported in Hertz (Hz). The abbreviations for the multiplicity of the proton, carbon and fluorine signals are as follows: s singlet, d doublet, dd doublet of doublets, ddd doublet of doublet of doublets, t triplet, dt doublet of triplets, q quartet, m multiplet, br s broad singlet.

Room temperature refers to 18-25 °C. Air and moisture sensitive reactions were carried out under an atmosphere of argon in oven-dried glassware. All evaporations and concentrations were performed under reduced pressure (*in vacuo*) by Büchi Rotavapor R-200. All reagents from Sigma Aldrich or Alfa Aesar were of synthetic grade and were used directly, without any further purification. When necessary, reagents were dried or purified prior to use according to standard methods.[1] Anhydrous solvents (DCM, THF, Et_2O) were obtained from MBraun MB SPS-800 solvent purification system by passage through two drying columns and dispensed under an argon atmosphere. Anhydrous MeOH and MeCN were distilled from calcium hydride in a recycling still.[1]

High resolution electrospray ionisation mass spectra were obtained on ThermoFisher Excalibur Orbitrap spectrometers operating in positive or negative mode, from solutions in MeOH or water, by the Mass Spectrometry Service at the University of St Andrews.

All microbiological works were carried out in a Gallenkamp laminar flowhood, using standard sterile techniques. Glassware and consumables for biological operations were sterilised by autoclaving, flaming or wiping with 75% ethanol before using. Sterilised consumables were used as supplied. Media were sterilised by 121 °C, 15 min autoclaving. Cell cultures were incubated in a temperature controlled incubator (New Brunswick Scientific). Centrifugation of 20 mL to 1 L was processed by Beckman

Avanti centrifuge. A hettich Mikro 200 bench-top centrifuge was used for micro-centrifugation.

All the primers were ordered from Eurogentec. Plasmids were sequenced by The MRC PPU DNA Sequencing and Services.

Growth of *Streptomyces calvus* on solid media

Streptomyces calvus was grown on solid ISP4 agar plates made by soluble starch (10 g), calcium carbonate (2 g), ammonium sulphate (2 g), sodium chloride (1 g), dipotassium phosphate (1 g), magnesium sulphate heptahydrate (1 g), ferrous sulphate (1 mg), manganese chloride (1 mg), zinc sulphate (1 mg), agar (20 g) and deionised water (to 1 L). The medium ISP4-agar was sterilised by autoclave before use. The plates were maintained at 30 °C incubator for 14 to 21 days. The spores were collected by means of sterilised cotton swabs and stored in a 25% glycerol solution at -80 °C. The petri plates after harvesting the spores can be stored at 4 °C for future use.

Seed culture of *Streptomyces calvus*

The seed culture was performed in TSBY liquid medium composed of 3% tryptone soy broth, 10.3% sucrose and 0.5% yeast extract. The seed culture of *S. calvus* was obtained by inoculating 50 μL spores into 50 mL TSBY, and the culture was allowed to grow at 28 °C for 2 days (250 mL conical flask shaking at 180 rpm).

Fermentation culture

A mass of the mycelium of *S. calvus* were obtained by inoculating a sterilised, defined medium (100 mL in 500 mL conical flask) with the seed culture obtained above (inoculate with 2 mL per 100 mL) and the culture was allowed to grow at 28 °C , 180 rpm for 8 days. The defined medium was composed of tap water (to 1 L), corn steep liquor (12.5 g), mannitol (10 g), sodium chloride (2 g), diammonium phosphate (2 g), monopotassium phosphate (1.5 g), magnesium sulphate heptahydrate (0.25 g), Hoagland's salt solution (1 mL), potassium fluoride solution (7.5 mL, 0.5 M).

Hoagland's salt solution contains deionised water (1 L), manganese(II) chloride tetrahydrate (0.389 g), phosphorous acid (0.611 g), copper(II) sulfate (0.056 g), ammonium molybdate tetrahydrate (0.056 g), nickel(II) sulfate hexahydrate (0.056 g), zinc sulfate heptahydrate (0.056 g), aluminum sulfate (0.056 g), stannous chloride dihydrate (0.028 g), cobalt(II) nitrate hexahydrate (0.056 g), titanium dioxide (0.056 g), lithium chloride (0.028 g), potassium iodide (0.028 g) and potassium bromide (0.028 g). Sterilised by autoclaving.

Extraction and purification of fluorometabolites

After 6 to 8 days incubation, the cells were discarded by centrifugation and the supernatant was extracted with charcoal/celite (5 g per 1000 mL). The charcoal/celite were mixed at a ratio of 1:2. The mixture, which was stirred in the supernatant for 1 hour and absorbed fluorometabolites, was collected by filtration and then washed by 100 mL acetone. The acetone was then dried *in vacuo*, and the residue was re-dissolved in deuterated oxide and analyzed by ^{19}F-NMR. The concentrated sample was then fractionated by HPLC. The fractions eluted at 14 to 15 min and 20 to 22 min were discretely collected. A further HPLC purification was then applied on each of these two fractions.

Genome sequencing

Streptomyces calvus strain T-3018 was cultured from the Pfizer culture collection. DNA for genome sequencing was isolated using Qiagen's DNA Maxi Kit according to the manufacturer's protocol. Specifications of the DNA submitted for sequencing was assessed on a NanoDrop spectrophotometer; Abs260/Abs280 = 1.87 and Abs260/Abs230 = 1.94. Sequencing was performed at the Beijing Genomics Institute (BGI) using an Illumina HiSeq 2000 platform. The genome sequence was determined to consist of 8,019,798 bp and G+C content was 72.39%. The Illumina data was assembled with SOAPdenovo into 70 contigs and 4 scaffolds (N50, 7,897,134). The genome sequence of T-3018 has been deposited to GenBank under accession number VCNP00000000.

Cloning and construction of His-tagged NucGT and NucGS overexpressing plasmid

Target genes were amplified by PCR from the genomic DNA of *S. calvus*. The gene *nucGT* was amplified with primers 5'-GAT ATC GGA TCC GAA TTC ATG CTG GTA AGC GTC GTC ACC CCC A-3' and 5'- AGT GCG GCC GCA AGC TTT CAG CCG CCG TTC AGG GCG GCG C-3'; nucGS was cloned with 5'- GAT ATC GGA TCC GAA TTC ATG GTC ACC GCA GCA CAG CAG A-3' and 5'- AGT GCG GCC GCA AGC TT T CAG GCG GCG GGC AGG ACG CCC-3'. His-tag was induced by primers at N-terminus. The plasmid pEHISTEV was digested with *EcoR*I and *Hind*III. The digested plasmid and fragments were purified by agarose gel and then recycled with QIAquick Gel Extraction Kit. The concentration of the vector and fragments were determined by Nanodrop. The recombination of target gene and vector were accomplished with Vazyme ClonExpress II One Step Cloning Kit. Chemically competent *E. coli* DH10B cells were independently transformed with recombinant plasmids and plated on LB agar containing kanamycin (50 μg/mL). The recombinant vectors were validated by colony PCR, double enzymatic digestion or DNA sequencing.

In-frame deletion of *nucGT* and *nucGS.*

Upstream and downstream sequences of the target genes were amplified by PCR from the genomic DNA of *S. calvus*. The upstream sequence of *nucGT* was amplified with primers 5'- ACG GCC AGT GCC AAG CTT TGA ACT GTG GGA GAC CAC GGC GGT -3' and 5'- ATC GTG CCA TTG AAA GGA CTG T AC CCG AAC TAC GGT GAG GAG CGC GT -3'; The downstream sequence of *nucGT* was amplified with primers: 5'- ACA GTC CTT TCA ATG GCA CGA TTC GGA CGC -3' and 5'- TGA CAT GAT TAC GAA TTC CGG TGA GTT CGA CGG TCT CCG CCG -3'. The upstream sequence of *nucGS* was amplified with primers 5'- ACG GCC AGT GCC AAG CTT GCT GGT CGC CGC TGG AAC CGA CGT GCA -3' and 5'- TCC ACC CCG AAA GGA CTT ACA ACC GAC GGA AGG GCT GCC A -3'; The downstream sequence of *nucGT* was amplified with primers: 5'- GTA AGT CCT TTC GGG GTG GAG GAG TGC GC -3' and 5'- TGA CAT GAT TAC GAA TTC TTC TTC GTC GTG TTC GCC GCG TTC AGC T -3'.

C112 fast clone kit provided by Vazyme was used to construct the knock-out vectors. The vectors were validated by PCR and DNA sequencing. The knock-out vectors were introduced into *S. calvus* by *E. coli*- Streptomyces conjugation. Single cross-over and double cross-over were screened and verified by PCR. The primers for Δ*nucGT* validation are: 5'- TGA CGT GCT CGA CGC GGT CGG TGC GGA -3' and 5'- CGC GAG CCC CAC GCG GCC CGT TCC CCT -3' (Figure S37). The primers for Δ*nucGS* validation are: 5'- AAG AGG CCG ACT TCT TCG AAC GGC A -3' and 5'-TTC TTC ATG CGG CAG TAC CTG TCC GA -3' (Figure S38).

The amino acid residues sequences of NucGT and NucGS.

NucGT (AMP46605.1):

VLVSVVTPTHNRPDRLKTALDSVRTLDFDGQLEVIVVNDNGTPVDDVVEAAARDLDVRLIDLPVNGGV
SAARNTGLEAAKGEYVAFLDDDDVFAPHHLTDTLPLLQSGADFVYNSVVIARTRVTGTTIDQAEVFVR
MDFPCDRELLDVTNHIPPTAVVCRSPRAADVWFDPTLMVQEDWDMWLRMIHKHGYTIAHQPRVSSV
VHRIPGVQSLTTVSSDDIAALKPYEDTWNLLTERWRVDSERVREVRRFMPVMYRMAYDMMNNGTPV
DFHYYERTVRVLYNALGDPNYGEERVVNEIRAALNGG

NucGS:

VVTAAQQTASAPDAARTFPKGFLWGSATASYQIEGAAAEDGRTPSIWDTYARTPGRVRNGDTGDVA
TDHYHRWREDVALMAELGLRAYRFSLAWPRIQPTGRGPAVQKGLDFYRRLVDELLDKGIQPVATLYH
WDLPQELEDAGGWPERATAERFAEYAALAADALGDRVKTWTTLNEPWCSAFLGYGSGVHAPGRTD
PVAALRAAHHLNLGHGLAVQALRDRIRADAQCSVTLNIHHVRPLTGSDGDADAVRRIDALANRVFTGP
MLRGAYPEDLFKDTTALTDWSFVRDGDLRQIQQPLDFLGVNYYSPTLVSESDGTGTHNADGHGRSA
HSPWPGSDQVSFHQPPGETTAMGWAVDPSGLYELLRRLSTDFPELPLVITENGAAFDDYADPAGQV
NDPARIAYLRGHLAAVHQAIADGSDVRGYFLWSLLDNFEWAHGYSKRFGAVYVDYPTGRRIPKASAR
WYSEVVRTGVLPAA

Protein overexpression and purification

Rosetta™(DE3) competent cells which contain pEHISTEV-*GT* or pEHISTEV-*GS* were used to overexpress the target protein. The cells were grown in 4 L LB medium supplemented with 25 mg/L (final concentration) kanamycin at 37 °C with shaking at

180 rpm until the absorbance at OD600 reached 0.6-0.8. At which point protein expression was induced by addition of 0.1 mM (final concentration) isopropyl β-D-1-thiogalactopyranoside (IPTG) with further incubation at 16 °C for 15 hours. The cells were harvested by centrifugation at 6000 rpm, 30 min, and re-suspended in 120 mL ice-cold binding buffer (50 mM Tris-HCl, pH 8.0, 10 mM imidazole, 1% Triton X-100, and 5% (V/V) glycerol), and disrupted by a Constant System Ltd. Cell Disruptor. Cellular debris was centrifugated at 12500 rpm for 30 min at 4 °C and discarded. The supernatant was filtered with a 0.22 μm filter membrane, and then loaded onto 2 mL Ni-NTA-affinity resin (GE Healthcare) pre-equilibrated with the binding buffer. The column was then washed with 10 mL washing buffer (50 mM Tris-HCl, pH 8.0, 45 mM imidazole, 300 mM NaCl). The protein was eluted with eluting buffer (50 mM Tris-HCl, pH 8.0, 250 mM imidazole). The fractions were collected and analyzed by SDS-PAGE (12% Tris-glycine gel) and the desired fractions were combined, and dialyzed in 4 L 20 mM Tris-HCl buffer. The protein solution was concentrated using an Centrifugal Filter Units (Milipore, 30,000 MWCO). The concentrated protein was flash frozen in liquid nitrogen, stored at −80 °C until further use. Protein concentration was determined by Nanodrop.

Enzymatic Reactions of NucGT or NucGS

NucGT assays were carried out in 50 mM Tris-HCl buffer, pH = 8.0, with 10 mg/mL UDP-glucose, 100 mM $MgCl_2$, 1 mM substrate and 1.5 nM glucosyltransferase. The reactions were incubated in a heatblock at 30 °C and samples were periodically collected every 30 min (for the first 3 hours) or 60 min. The enzyme was denaturized by adding chloroform and the reaction monitored by HPLC, the identity of the products were confirmed by MS of isolated fractions.

NucGS NMR assays were carried out in 50 mM Tris-HCl buffer, pH = 7.2, containing 10 percent deuterium oxide (D_2O), with approximately 1.5 μM F-Met **I** or **II** and 2.0 nM *β*-Glucosidase. The reactions were incubated in an oven incubator at 30 °C and analyzed with ^{19}F NMR every hour. Another NucGS assay was prepared to prove the production of 4-fluoro-adenosine **19**, which contained 80 nM *β*-Glucosidase under the same conditions, and was analysed with both ^{19}F NMR and LC-MS.

HPLC and LC-MS Parameters of Enzymatic Reaction

HPLC analysis was performed on a Shimazu LC-20-AT HPLC system. The samples were separated using a Phenomenex Luna C-18 Semi-prep column. LC-MS analysis was performed on a Thermo UltiMate 3000 HPLC system coupled in Diode Array detector and LCQ Fleet mass spectrometer in ESI positive mode using the column indicated in the individual experiment.

For the purification of F-Mets **I** and **II**, a Phenomenex C-18 Luna Semi-preparation column was used. F-Met **I** and a mixture of F-Met **II** and nucleocidin were co-purified with gradient elution: 0-5 min,100% mobile phase A (miliQ water, 0.05% TFA); 18 min, 95% mobile phase B (acetonitrile, 0.05% TFA); 23 min, 100% B; 25 min, 100% A; 35 min, 100% A. Then the mixture of F-Met **II** and nucleocidin were separated under isocratic elution: 10% B, 90% A, 15 min.

HPLC and LC-MS analysis of F-Met I, F-Met II and nucleocidin.

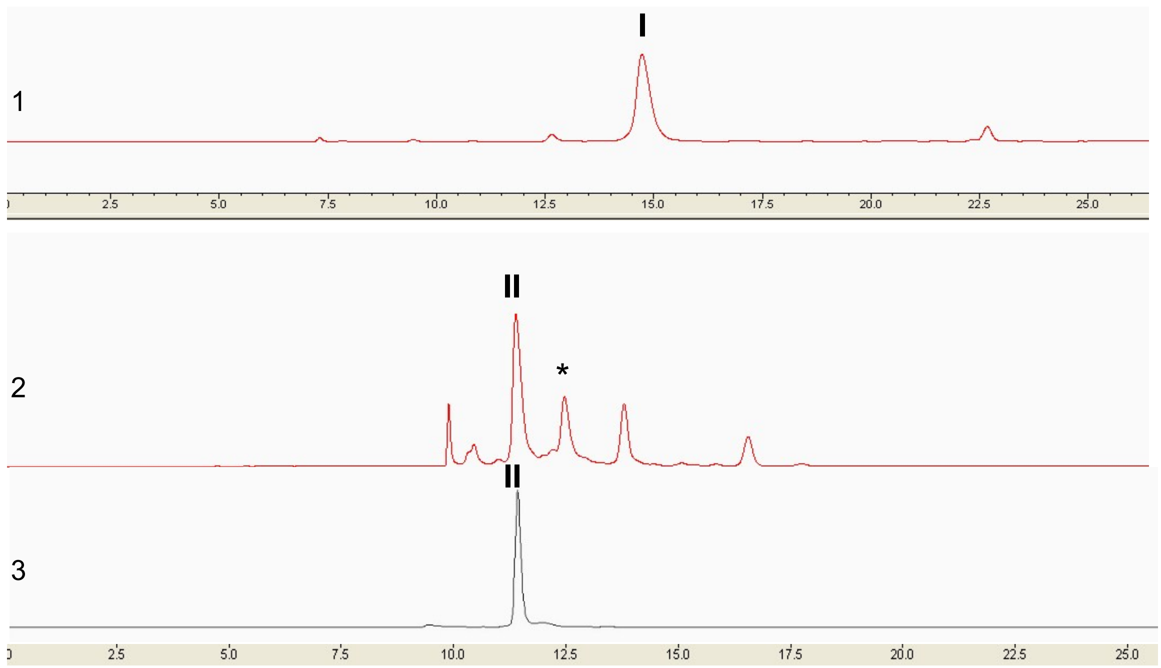

Figure S1 HPLC traces of purified compounds. (1) Purified F-Met **I** by gradient elution (Retention time = 14.9 min). (2) A further purification by isocratic elution of the mixture of F-Met **II** and nucleocidin (*) obtained from gradient elution semi-prep (retention time = 21 to 22 min). (3) Purified F-Met **II** (11.8 min).

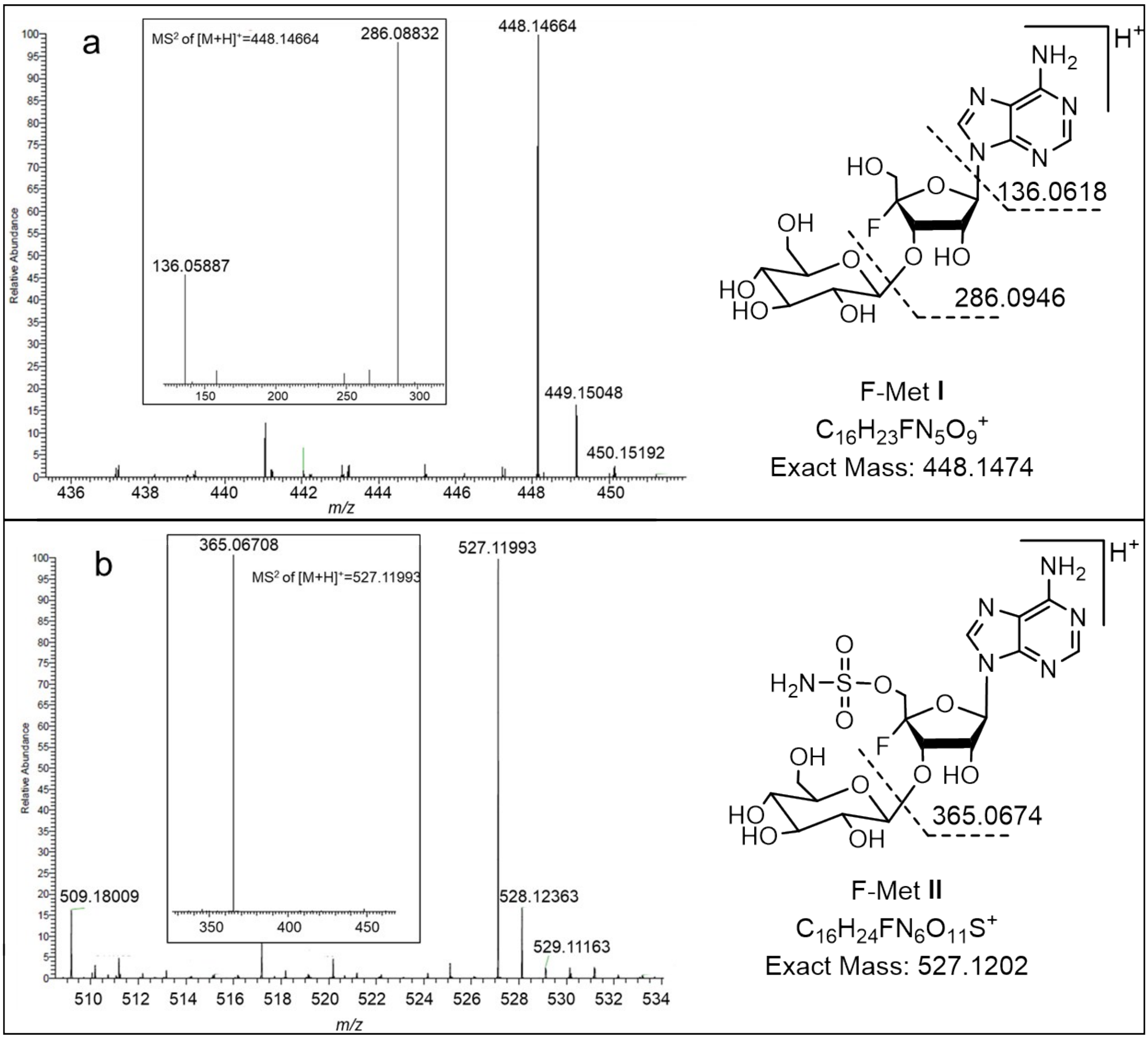

Figure S2 LC-HRMS and MS^2 analysis of F-Mets **I** and **II**. **(a)** For F-Met **I**, the calculated $[M+H]^+$ = 448.1474, the experimental $[M+H]^+$ = 448.14664, error = -1.7 ppm; **(b)** The calculated $[M+H]^+$ of F-Met **II** is 527.1202, the experimental $[M+H]^+$ = 527.11993, error = -0.5 ppm.

Nuclear magnetic resonance spectroscopy analysis of F-Met I and F-Met II.

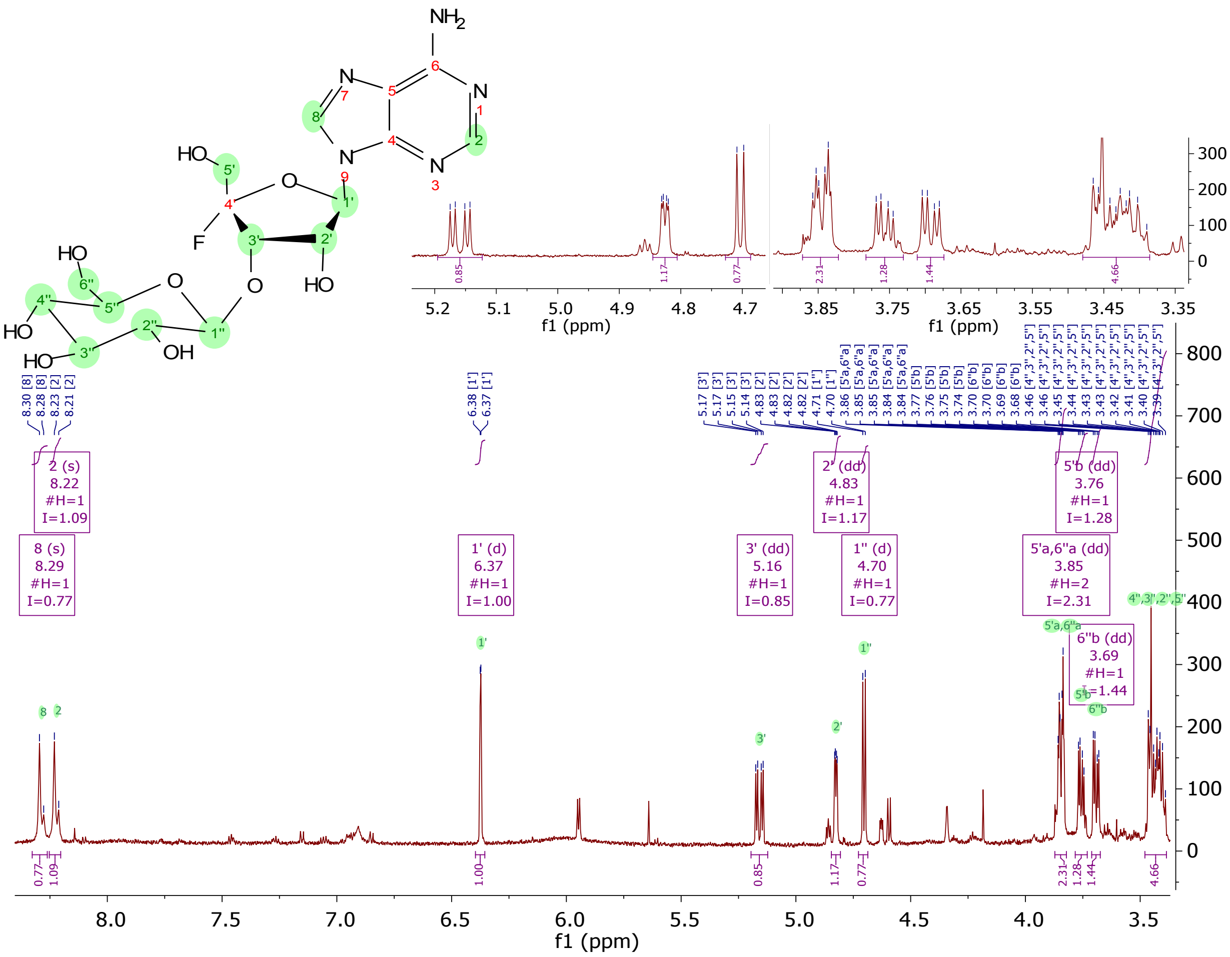

Figure S3. ^{1}H NMR of F-Met **I** (700 MHz, Acetone-d_6).

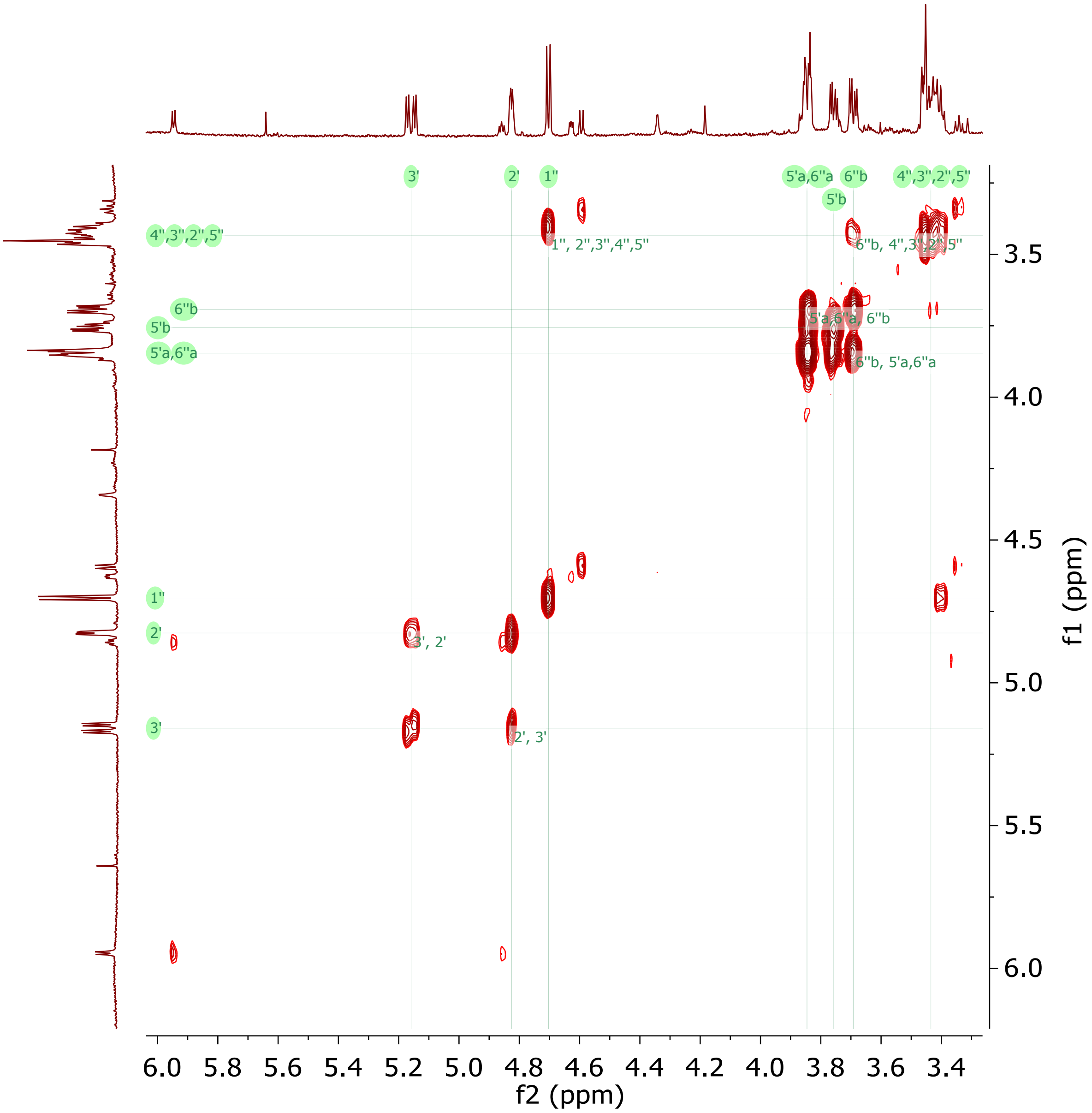

Figure S4. ^1H-^1H COSY of F-Met **I** (700 MHz, Acetone-d_6).

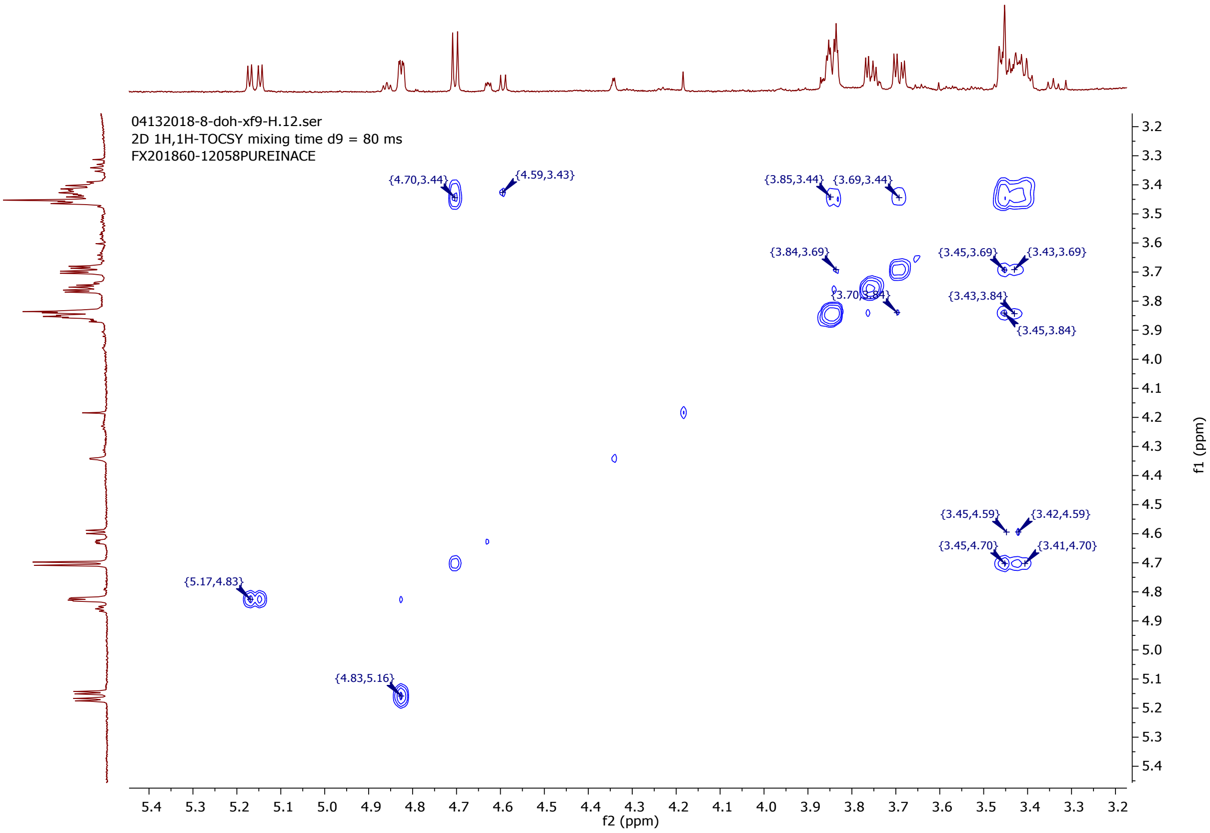

Figure S5. ^1H-^1H TOCSY of F-Met **I** (700 MHz, Acetone-d_6).

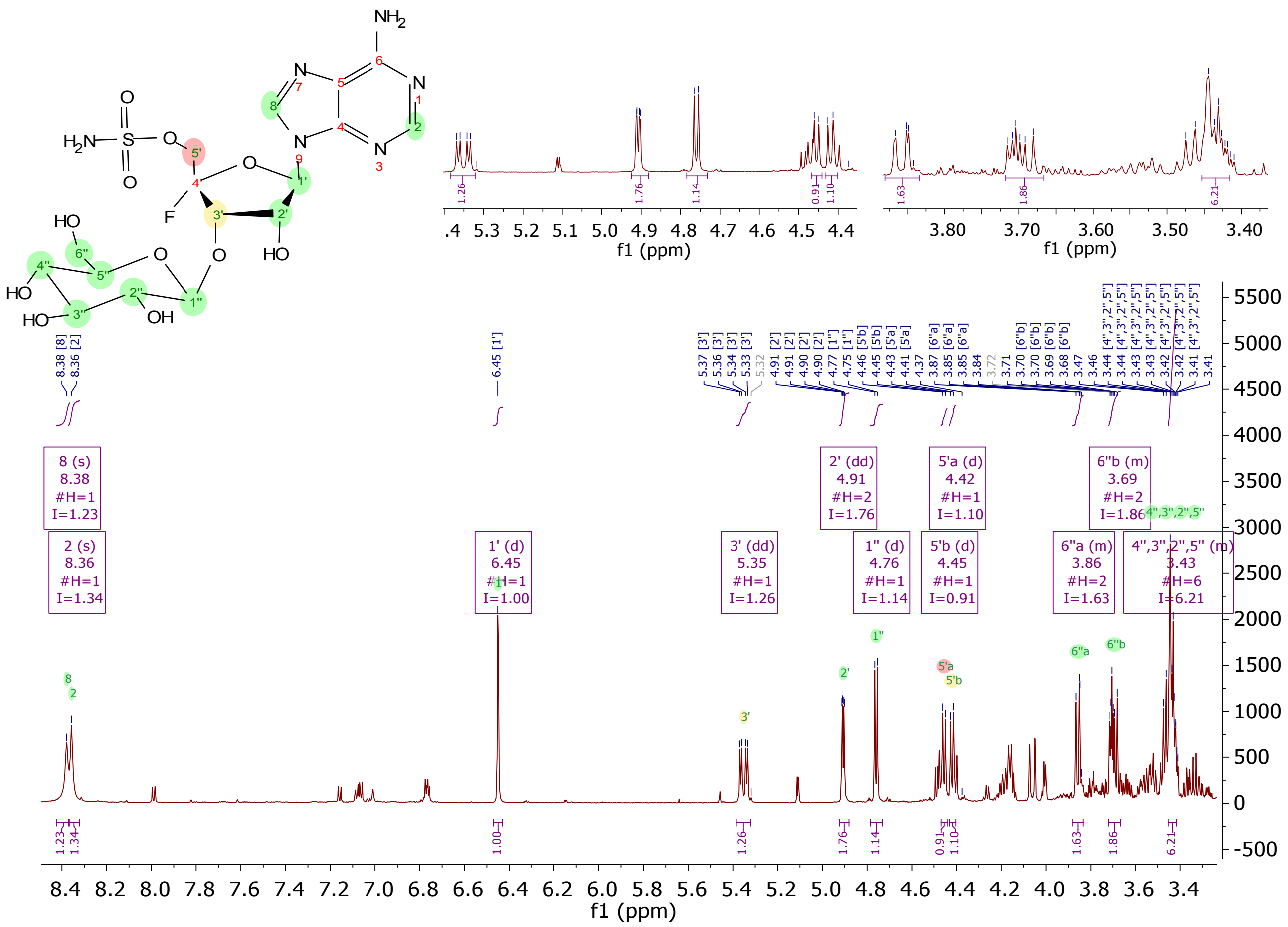

Figure S6. ^{1}H NMR of F-Met **II** (700 MHz, Acetone-d_6).

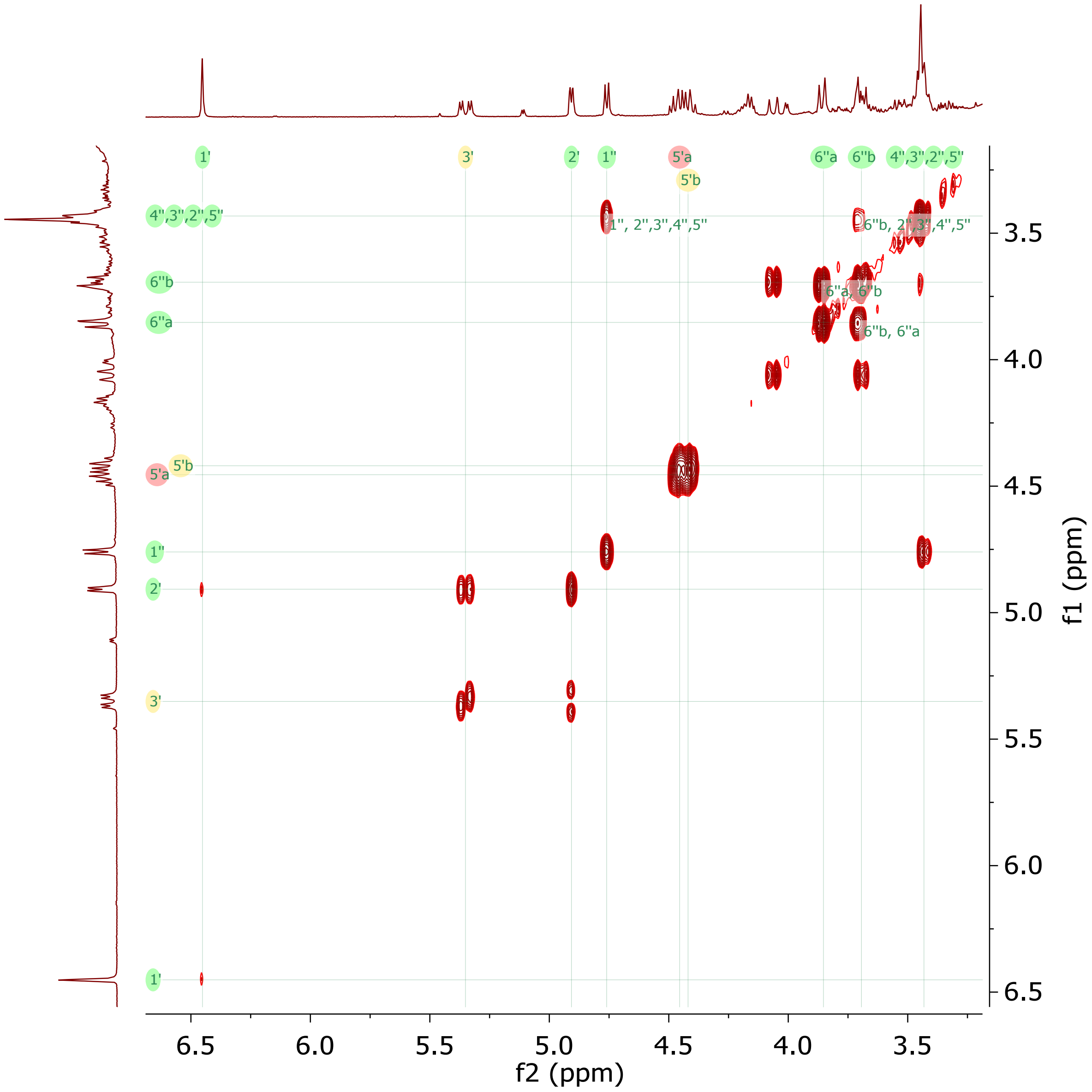

Figure S7. ^{1}H-^{1}H COSY of F-Met **II** (700 MHz, Acetone-d_6).

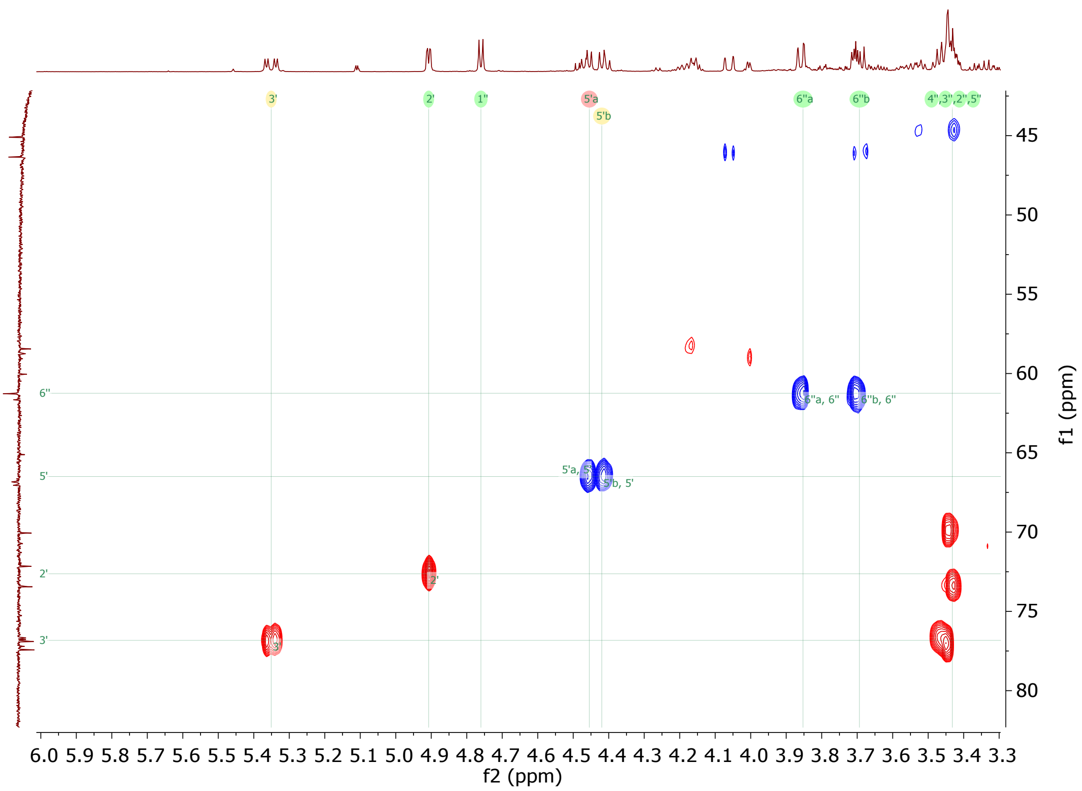

Figure S8. ^{1}H-^{13}C HSQC of F-Met **II** (700 MHz, Acetone-d_6).

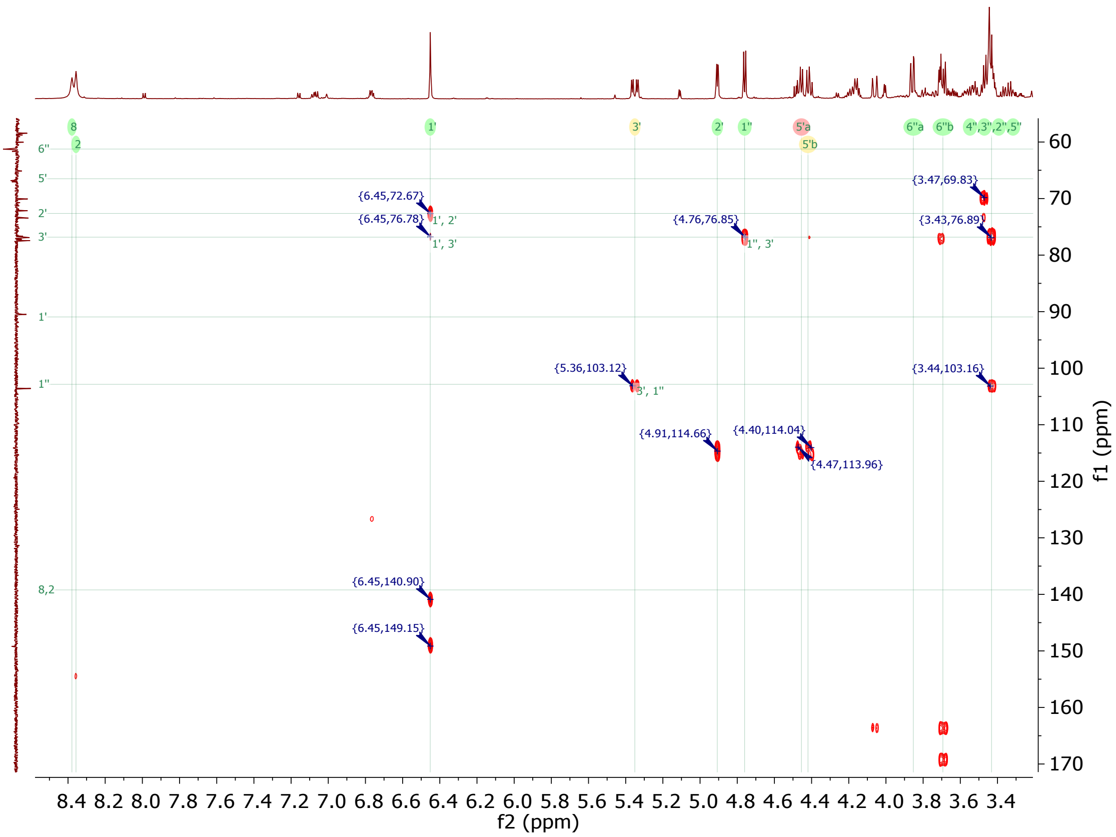

Figure S9. ^{1}H-^{13}C HMBC of F-Met **II** (700 MHz, Acetone-d_6).

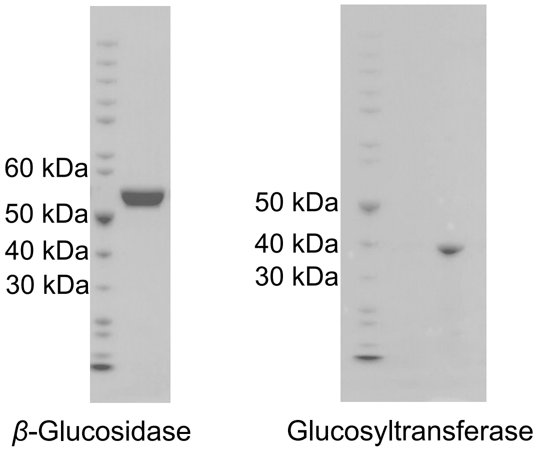

Figure S10. SDS-PAGE analysis of the purified proteins NucGS and NucGT

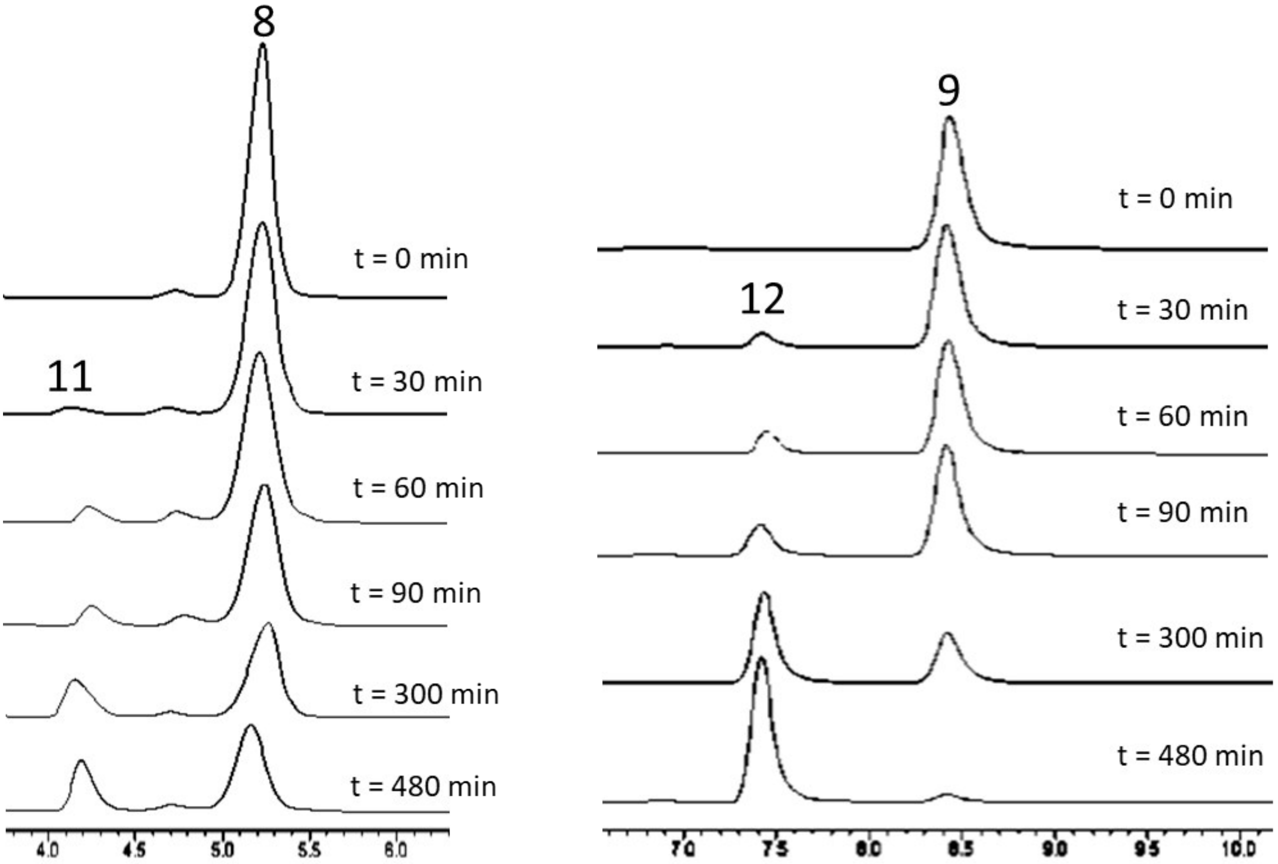

Figure S11. Glucosyltransferase assays with adenosine **8** or compound **9** and the generation of compound **11** or **12**.

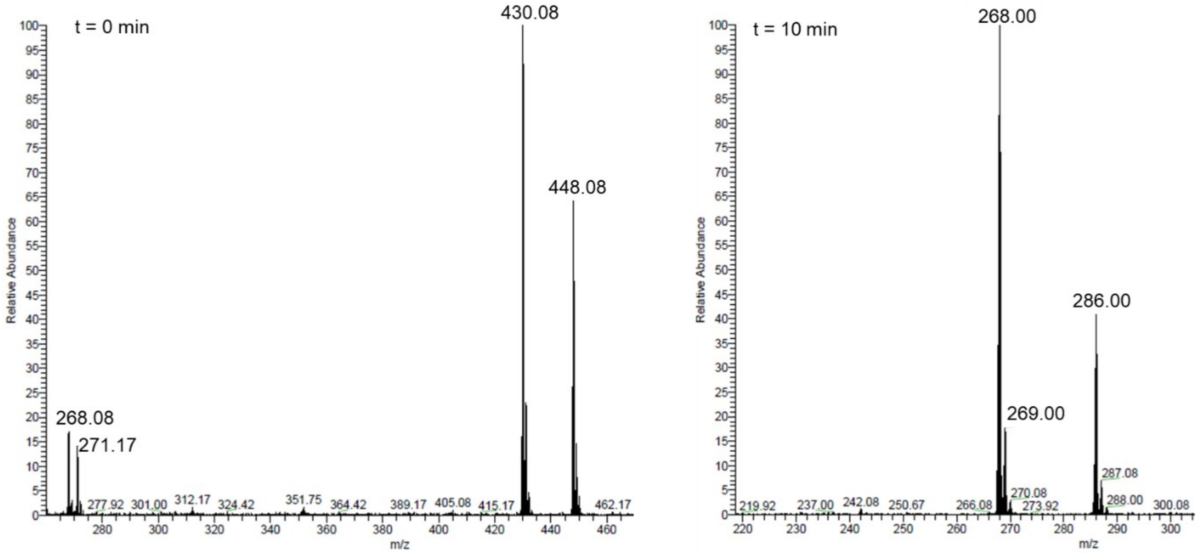

Figure S12. LC-MS analysis of *β*-Glucosidase (2 mg) reaction with a mixture of F-Met **I** and compound **11**.

Compound preparation

Sulfamoyladenosine (9)

Sulfamoyladenosine **9** was synthesised using previously reported methods[2]; 1H NMR (400 MHz, D_2O) δ 8.44 (s, 1H, Ade H-2), δ 8.40 (s, 1H, Ade H-8), δ 6.15 (d, *J*=4.5, 1H, ribose H-1'), 4.53 (d, *J*=4.9, 1H, ribose H-2'),4.45-4.42 (4H, m, ribose H-3' & H-4' & H-5'); HRMS calculated for $C_{10}H_{15}N_6O_6S$ [M+H]+ 347.0768, found 347.0761. Data are in agreement with the literature.[2]

Adenosine-5'-sulfate (10)

Adenosine-5'-sulfate **10** was synthesised using previously reported methods [3] 1H NMR (400 MHz, D_2O) δ 8.42 (s, 1H, Ade H-2), δ 8.39 (s, 1H, Ade H-8), δ 6.08 (d, *J*=5.1, 1H, ribose H-1'), 4.73 – 4.72 (m, 1H, ribose H-2'), 4.43 (dd, *J*=4.6, 4.4, 1H, ribose H-3'), 4.35 – 4.32 (m, 1H, ribose H-4'), 4.28 – 4.13 (m, 2H ribose H-5'); HRMS calculated for $C_{10}H_{12}N_5O_7S$ [M-H]- 346.0457, found 346.0458. Data are in agreement with the literature.[3]

5-*O*-Tert-butyldiphenylsilyl-1,2-*O*-Isopropylidene-α-D-xylofuranose

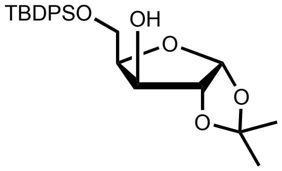

1,2-*O*-Isopropylidene-α-D-xylofuranose (5.0 g, 26.29 mmol, 1.0 equiv) was dissolved in dry *N,N*-dimethylformamide (100 mL) and the resulting mixture cooled to 0 ºC. Imidazole (5.0 g, 73.61 mmol, 2.8 equiv) was added, followed by *tert*-butyl-diphenylsilyl chloride (8.96 mL, 34.44, 1.3 equiv) dropwise over 20 min. The mixture was stirred at 0 ºC for 20 min and for a further 2 hours at room temperature. The mixture was then concentrated under reduced pressure, diluted with ethyl acetate (50 mL) and washed with a saturated aqueous solution of ammonium chloride (30 mL), saturated sodium hydrogen carbonate (30 mL), saturated brine (30 mL), dried onto magnesium sulfate, filtered and concentrated under reduced pressure to afford a solid residue. Purification by silica flash chromatography, eluting with a 10/90 to 20/80 mixture of ethyl acetate/hexane, gave the desired compound as a colourless solid (9.16 g, 81% yield). ^{1}H NMR (500 MHz, $CDCl_3$) NMR (500 MHz,*J*=6.3, 2H), 7.67 (d,

J=6.3, 2H), 7.48 – 7.37 (m, 2H), 6.01 (d, *J*=3.7, 1H), 4.55 (d, *J*=3.7, 1H), 4.37 (t, *J*=2.8, 1H), 4.17 – 4.06 (m, 4H), 1.47 (s, 3H), 1.33 (s, 3H), 1.05 (s, 9H). These data are in good agreement with the literature values.[4]

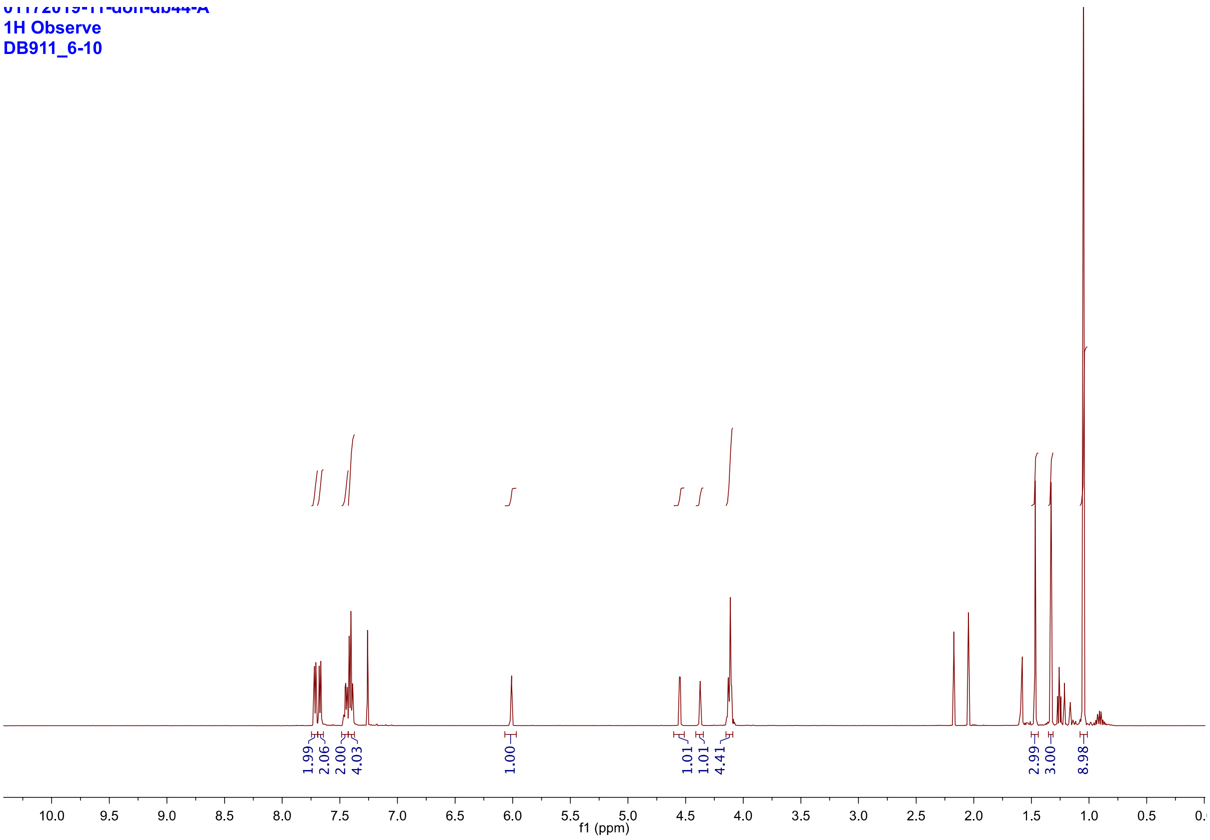

Figure S13. ^{1}H NMR of compound 5-O-Tert-butyldiphenylsilyl-1,2-O-Isopropylidene-α-D-xylofuranose

5-*O*-Tert-butyldiphenylsilyl-1,2-*O*-isopropylidene-α-D-xylo-3-ulose

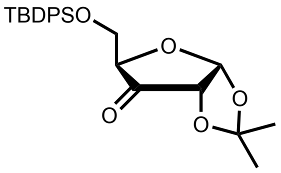

Oxalyl chloride (2.05 mL, 23.46 mmol, 1.1 equiv) was dissolved in dry dichloromethane (60 mL) under an atmosphere of argon at - 60 °C. Dimethylsulfoxide (3.3 mL, 46.91 mmol, 2.2 equiv) was added, followed by 5-*O*-tert-butyldiphenylsilyl-1,2-*O*-Isopropylidene- *α* -D-xylofuranose (9.14 g, 21.32 mmol, 1.0 equiv) dissolved in 20 mL of dry dichloromethane, dropwise. The resulting mixture was stirred at -60 °C for 30

min, then triethylamine (9.75 mL, 70.36 mmol, 3.3 equiv) was added and the mixture stirred at -60 °C for 1 hour, then allowed to warm to room temperature and stirred for a further 3 hours. The mixture was then poured onto 100 mL of ice cold water, the layers separated, the aqueous layer extracted with dichloromethane (2 x 20 mL) and the combined organic layers washed with a saturated aqueous solution of sodium hydrogen bicarbonate (30 mL), brine (30 mL), dried onto magnesium sulfate, filtered and concentrated under reduced pressure to furnish a colourless solid, which was used in the next synthetic step without any further purification (9.1 g, quantitative yield). ^{1}H NMR (500 MHz, $CDCl_3$) δ = 7.69 – 7.66 (m, 2H), 7.61 – 7.59 (m, 2H), 7.41 (m, 6H), 6.26 (d, *J*=4.5, 1H), 4.43 (dd, *J*=4.5, 1.1, 1H), 4.39 (td, *J*=2.0, 1.0, 1H), 3.91 (dd, *J*=11.1, 1.9, 1H), 3.85 (dd, *J*=11.1, 2.2, 1H), 1.48 (s, 3H), 1.47 (s, 3H), 1.00 (s, 9H). These data are in good agreement with the literature values.[4]

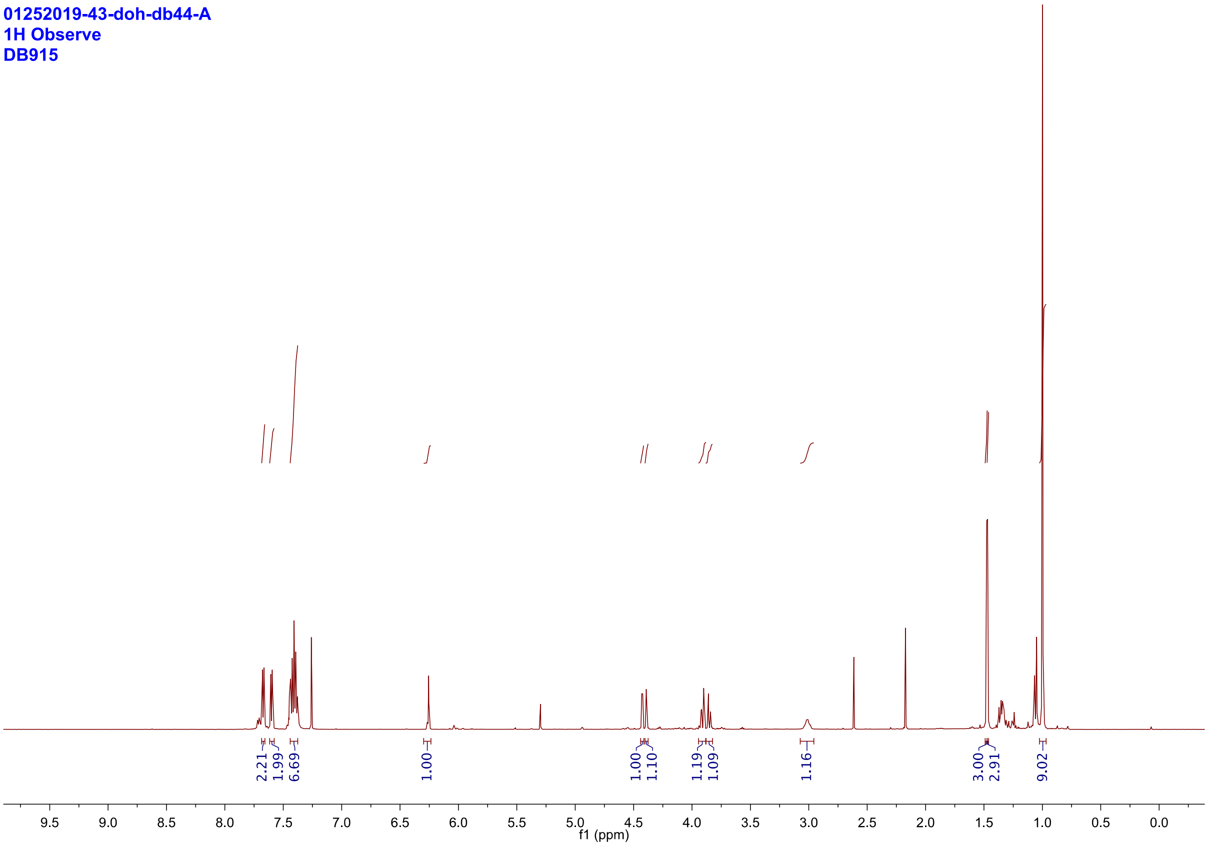

Figure S14. ^{1}H NMR of 5-O-Tert-butyldiphenylsilyl-1,2-O-isopropylidene-α-D-xylo-3-ulose

5-*O*-Tert-butyldiphenylsilyl-1,2-*O*-Isopropylidene-*α*-D-ribofuranose 13

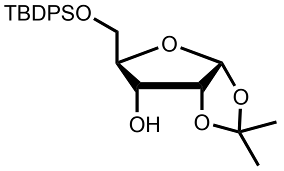

5-*O*-Tert-butyldiphenylsilyl-1,2-*O*-isopropylidene-*α*-D-xylo-3-ulose (9.1 g, 21.32 mmol, 1.0 equiv) was dissolved in a 80/20 mixture of ethanol and water (200 mL) and the resulting mixture cooled to 0 °C. Sodium borohydride (5.2 g, 138.58 mmol, 6.5 equiv) was added in four portions over 30 min. The resulting mixture was stirred at 0 °C for 3 hours, then poured onto ice cold water (350 mL). The aqueous layer was extracted with ethyl acetate (5 x 60 mL), the combined organic layers dried onto magnesium sulfate, filtered and concentrated to give a solid residue. Purification by silica flash chromatography, eluting with 20/80 ethyl acetate/hexane, furnished the title compound as a colourless solid (6.84 g, 75% yield). ^{1}H NMR (500 MHz, $CDCl_3$) δ = 7.72 – 7.66 (m, 4H), 7.44 – 7.35 (m, 6H), 5.85 (d, *J*=3.8, 1H), 4.60 (dd, *J*=5.2, 3.8, 1H), 4.20 – 4.10 (m, 1H), 3.98 – 3.93 (m, 1H), 3.88 – 3.82 (m, 1H), 1.56 (s, 3H), 1.41 – 1.34 (m, 3H), 1.05 (s, 9H). These data are in good agreement with the literature values.[4]

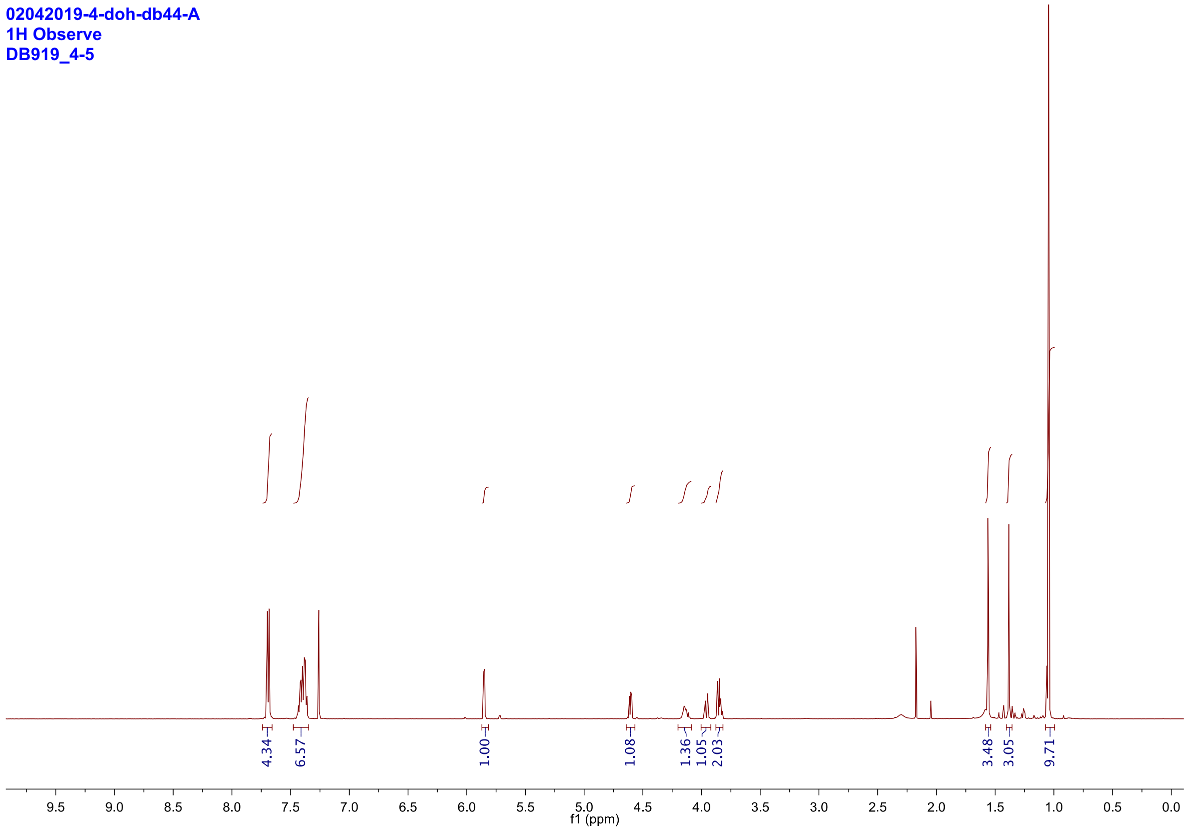

Figure S15. ^{1}H NMR of compound **13**

1,2,3,4,6-Penta-*O*-benzoyl-*α*-D-glucopyranoside

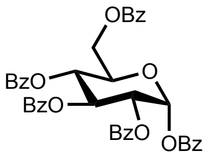

D-glucopyranose (10.0 g, 55.5 mmol, 1.0 equiv) was dissolved in a mixture of dry pyridine (40 mL) and dry dichloromethane (80 mL) under an atmosphere of argon. The mixture was cooled to -20 ºC and benzoyl chloride (40 mL, 344.3 mmol, 6.2 equiv) was added dropwise over a period of 10 min. The resulting mixture was allowed to warm to 0 ºC and stirred for a further 30 min, then it was left standing in a fridge at 4 ºC for 16 hours. The mixture was then stirred at room temperature for a further 5 hours, and crushed ice (150 mL) and dichloromethane (200 mL) were added. The layers were separated and the organic layer was washed with a 3.0 M aqueous solution of sulfuric acid (2 x 100 mL), saturated sodium hydrogen carbonate (2 x 100 mL), brine (2 x 100 mL), dried onto magnesium sulfate, filtered and concentrated under reduced pressure to afford a thick colourless material. This was dissolved in a hot 70/30 mixture of ethanol and water, and the resulting mixture allowed to cool down to room temperature and left standing for 16 hours, during which time a colourless solid crashed out of the solution. The resulting material was filtered and dried under reduced pressure, furnishing the desired compound as a colourless solid (29.0 g, 75% yield). ^{1}H NMR (500 MHz, $CDCl_3$) δ = 8.23 – 8.15 (m, 2H), 8.08 – 8.02 (m, 2H), 7.99 – 7.95 (m, 2H), 7.94 - 7.88 (m, 4H), 7.72 – 7.66 (m, 1H), 7.61 – 7.42 (m, 8H), 7.41 – 7.37 (m, 2H), 7.36 - 7.30 (m, 4H), 6.88 (d, *J*=3.7, 1H), 6.35 (t, *J*=10.0, 1H), 5.89 (t, *J*=9.9, 1H), 5.71 (dd, *J*=10.3, 3.8, 1H), 4.70 – 4.59 (m, 2H), 4.55 – 4.46 (m, 1H). These data are in good agreement with the literature values.[5]

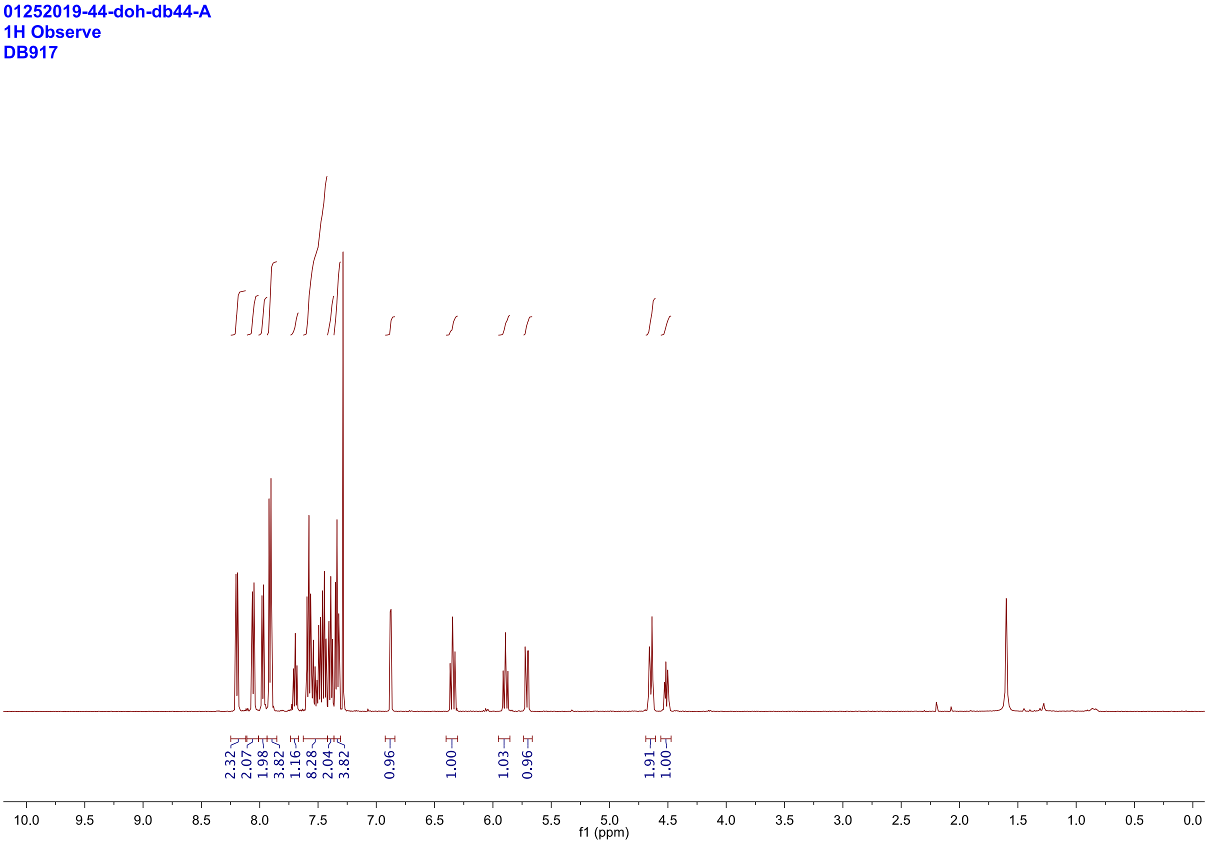

Figure S16. ^{1}H NMR of 1,2,3,4,6-Penta-O-benzoyl-α-D-glucopyranoside

2,3,4,6-Tetra-*O*-benzoyl-D-glucopyranose

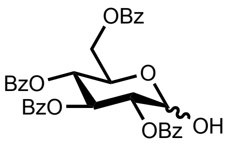

2,3,4,6-Tetra-O-benzoyl-D-glucopyranose was synthesised using a modified procedure developed from the method reported by Murakami *et al*.[6] Perbenzoylated-β-D-glucose (8.0 g, 12.9 mmol, 1.1 equiv) was dissolved in dry dichloromethane (120 mL) under an atmosphere of argon. Hexamethyldisilane (1.6 mL, 7.76 mmol, 0.6 equiv) was added, followed by zinc iodide (1.03 g, 3.22 mmol, 0.25 equiv). The pink resulting mixture was stirred for a few minutes, and then iodine (2.0 g, 7.76 mmol, 0.6 equiv) was added in one portion and the resulting brown mixture stirred for a further 2 hours, or until the colour turned to a faint pink. An 1.0 M sodium hydrogen carbonate aqueous solution containing 10% of sodium thiosulfate (20 mL or until the mixture turned clear) was added, the layers separated and the organic layer washed with brine (2 x 50 mL), dried onto magnesium sulfate, filtered and concentrated under reduced

pressure to afford a solid residue. This material was dissolved in acetone (80 mL) and water (3 mL) was added, followed by silver carbonate (1.8 g, 6.45 mmol, 0.5 equiv). The mixture was stirred at room temperature for 2 hours or until the starting material had disappeared and more polar compounds had formed. The mixture was filtered onto Celite, the filtrate concentrated under reduced pressure, and the resulting residue re-dissolved in dichloromethane (80 mL), dried onto magnesium sulfate, filtered and concentrated under reduced pressure to afford a solid residue. Purification by silica flash chromatography, eluting with a 30/70 mixture of ethyl acetate and hexane, afforded the title compound as a colourless solid (6.63 g, 86% yield). This material was believed to consist of an inseparable mixture of the *α* and *β* anomers, due to the broad signal at 3.15 ppm assigned to the anomeric proton.

^{1}H NMR (500 MHz, $CDCl_3$) δ = 8.06 (d, *J*=8.0, 2H), 7.99 (d, *J*=7.8, 2H), 7.94 (d, *J*=7.8, 2H), 7.88 (d, *J*=7.6, 2H), 7.58 – 7.49 (m, 3H), 7.45 – 7.34 (m, 7H), 7.30 (t, *J*=7.7, 2H), 6.25 (t, *J*=9.9, 1H), 5.81 – 5.68 (m, 2H), 5.32 (dd, *J*=10.3, 3.6, 1H), 4.73 – 4.62 (m, 2H), 4.45 (dd, *J*=12.0, 4.2, 1H), 3.15 (br s, 1H). These data are in good agreement with the literature values.[5]

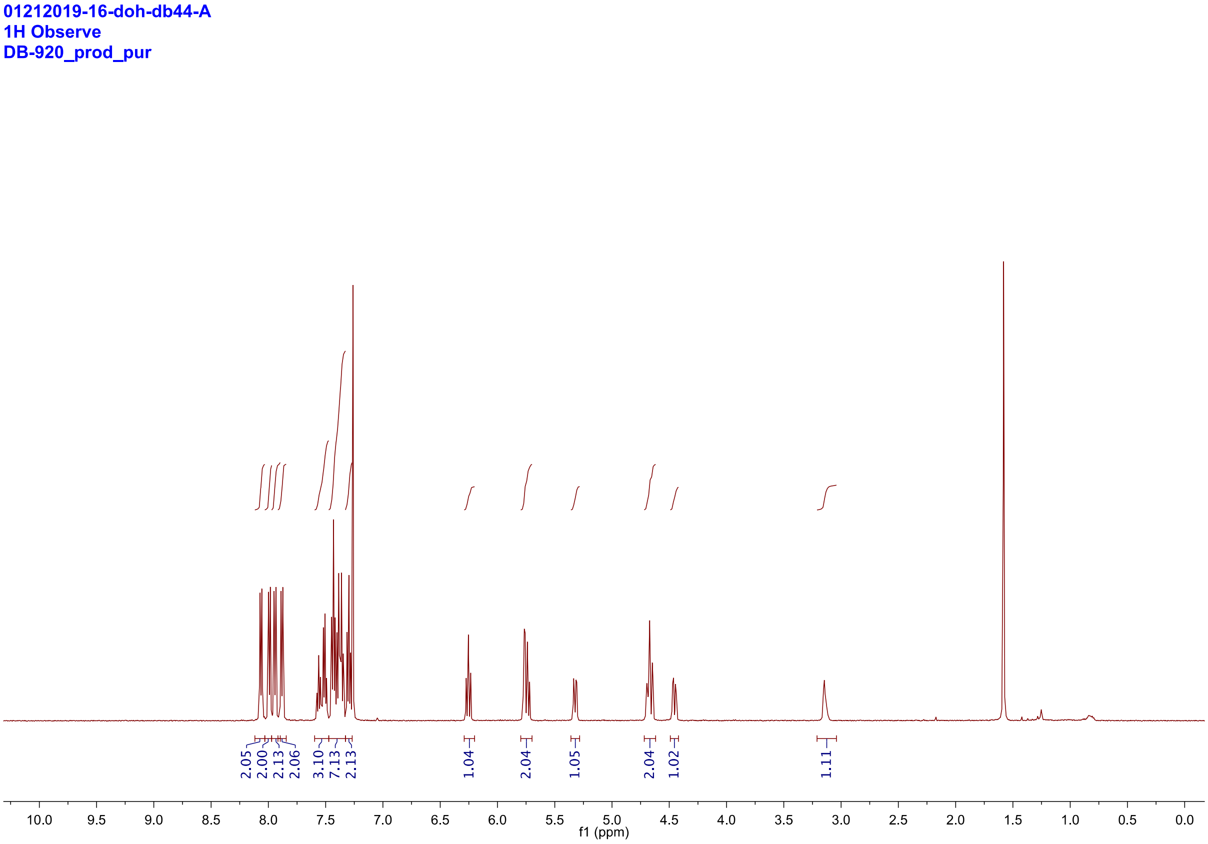

Figure S17. ^{1}H NMR of 2,3,4,6-Tetra-O-benzoyl-D-glucopyranose

2,3,4,6-Tetra-O-benzoyl-1-(2,2,2-trichloroethanimidate)- α -D-glucopyranoside 14

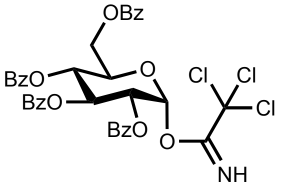

2,3,4,6-Tetra-*O*-benzoyl-D-glucopyranose (6.63 g, 11.0 mmol, 1.0 equiv) was dissolved in dry dichloromethane under an atmosphere of argon. Trichloroacetonitrile (11.0 mL, 110.0 mmol, 10.0 equiv) was added, followed by 1,8-diazabicyclo(5.4.0)undec-7-ene (489 μL, 3.3 mmol, 0.3 equiv) and the resulting mixture was stirred at room temperature for 4 hours. The mixture turned to a deep dark brown colour, and it was concentrated under reduced pressure and the residue purified by silica flash column chromatography, eluting with 20/80 ethyl acetate/hexane, to afford the desired compound as a light brown solid (5.31 g, 65% yield). ^{1}H NMR (700 MHz, $CDCl_3$) δ = 8.63 (s, 1H), 8.06 – 8.02 (m, 2H), 7.98 – 7.93 (m, 4H), 7.89 – 7.84 (m, 2H), 7.58 – 7.54 (m, 1H), 7.53 – 7.50 (m, 2H), 7.46 – 7.41 (m, 3H), 7.37 (t, *J*=7.9, 5H), 7.32 – 7.29 (m, 2H), 6.84 (d, *J*=3.7, 1H), 6.27 (t, *J*=10.0, 1H), 5.82 (t, *J*=10.0, 1H), 5.62 (dd, *J*=10.2, 3.7, 1H), 4.68-4.62 (m, 2H), 4.51 – 4.46 (m, 1H). These data are in good agreement with the literature values.[5]

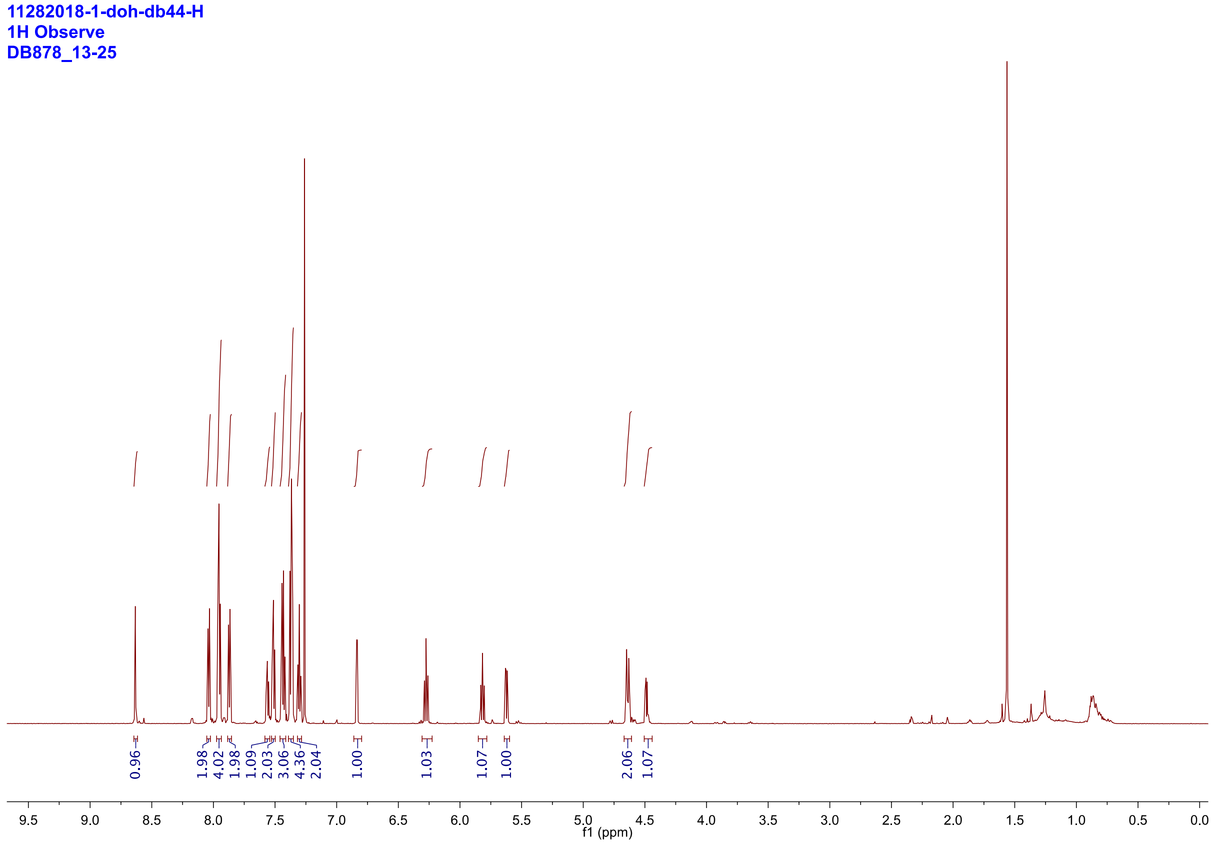

Figure S18. ^{1}H NMR of compound **14**

5-*O*-Tert-butyldiphenylsilyl-3-*O*-(β -D-2',3',4',6'-tetra-*O*-benzoylglucopyranosyl)-1,2-*O*-isopropylidene-a-l-ribo-furanoside 15

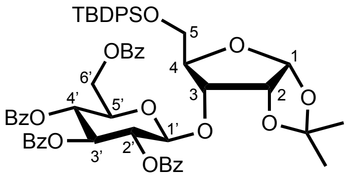

2,3,4,6-Tetra-O-benzoyl-1-(2,2,2-trichloroethanimidate)- α -D-glucopyranoside **14** (1.98 g, 2.7 mmol, 1,1 equiv) and 5-*O*-tert-butyldiphenylsilyl-1,2-*O*-Isopropylidene- α -D-ribofuranose **13** (1.04 g, 2.43 mmol, 1.0 equiv) were dissolved in dry dichloromethane (20 mL) under an atmosphere of argon. Molecular sieves (4Å, 500 mg) were added and the mixture stirred at room temperature for 15 min. Trimethylsilyl trifluoromethanesulfonate (44 µL, 0.24 mmol, 0.1 equiv) was added and the mixture stirred at room temperature for 2 hours. Triethylamine (0.5 mL) was then added, the mixture filtered and the filtrate concentrated under reduced pressure, to furnish a solid residue. Purification by silica flash chromatography, eluting with a 30/70 mixture of

ethyl acetate and hexane, gave the title compound as a colourless solid (1.22 g, 50% yield). A very pure sample was obtained by preparative HPLC purification using a Phenomenex C18 Luna 250 x 21.2 mm column with TMS endcapping, eluting with a 97/3 mixture of acetonitrile and methanol, flow 10 mL/min, 254 nm; Rt = 13.7 min; mp: 65-67 ºC; ^{1}H NMR (500 MHz, $CDCl_3$) NMR (500 MHz, CDCln mixture of acetonitrile and methanoleduced pressure, to furnish a solid residue. Purification by silica flash chromatogr*J*=7.8, 2H), 7.24 (t, *J*=7.8, 2H), 5.85 (t, *J*=9.6, 1H, *H-3'*), 5.71 – 5.63 (m, 2H, *H-2'* + *H-4'*), 5.61 (d, *J*=3.4, 1H, *H-1*), 4.97 (d, *J*=7.9, 1H, *H-1'*), 4.66 – 4.61 (m, 2H, *H-6'a* + *H-2*), 4.55 (dd, *J*=12.1, 5.8, 1H, *H-6'b*), 4.33 (dd, *J*=9.1, 4.3, 1H, *H-3*), 4.10 (m, 1H, *H-5'*), 3.93 – 3.88 (m, 2H, *H-4* + *H-5a*), 3.61 (dd, *J* = 12.2, 2.3 Hz, 1H, *H-5b*) 1.48 (s, 3H), 1.28 (s, 3H), 1.05 (s, 9H, *t*-Bu); ^{13}C NMR (126 MHz, $CDCl_3$) δ = 166.0, 165.8, 165.2, 164.7, 135.7, 135.5, 133.6, 133.5, 133.3, 133.2, 132.8, 130.0, 129.9, 129.8, 129.7, 129.6, 129.5, 129.0, 128.8, 128.7, 128.5, 128.4, 128.4, 128.3, 128.0, 127.9, 113.2, 103.4, 101.8, 79.3, 78.22, 77.4, 73.0, 72.4, 71.7, 69.7, 63.1, 60.6, 26.9, 26.77, 26.3, 19.5; *m/z* (ESI$^+$): Found $[M+Na]^+$ 1029.3464. $C_{58}H_{58}NaO_{14}Si$ requires M^+, 1029.3494.

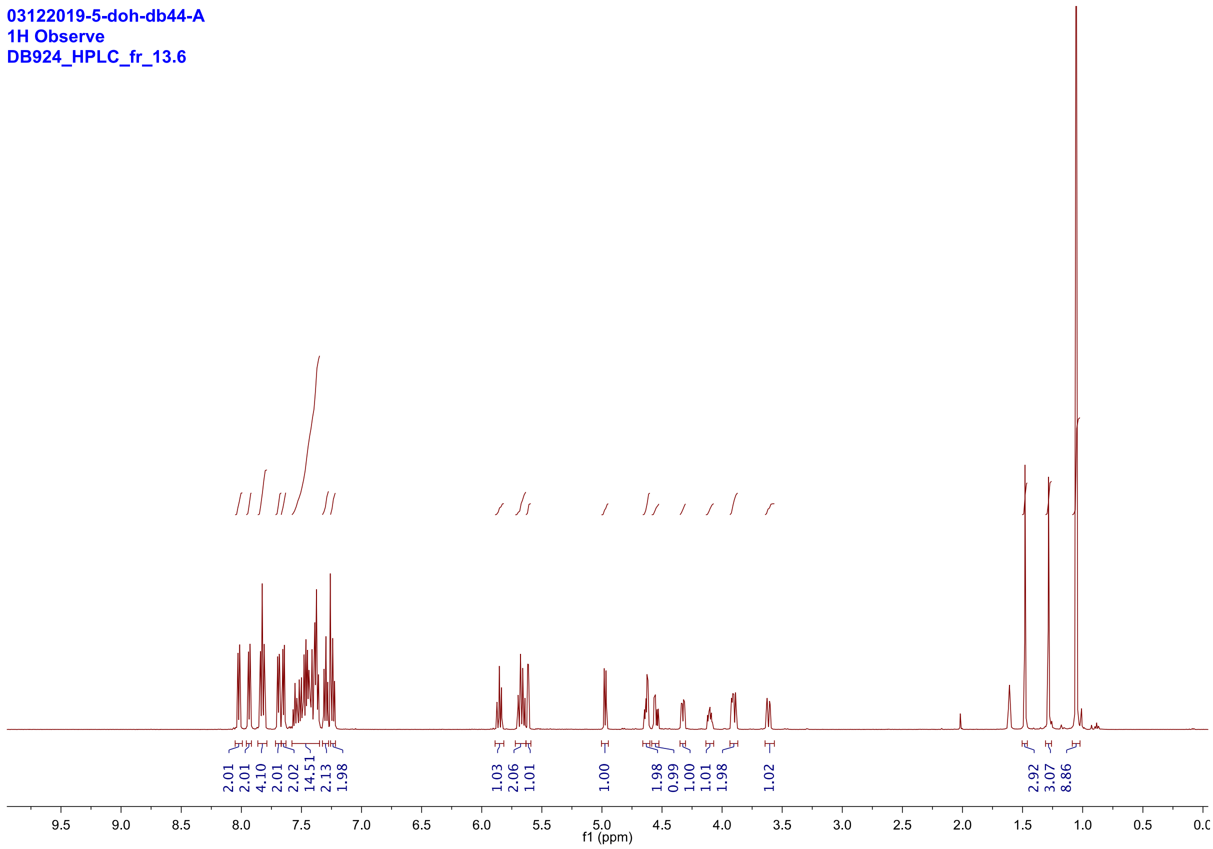

Figure S19. ^{1}H NMR of compound **15**

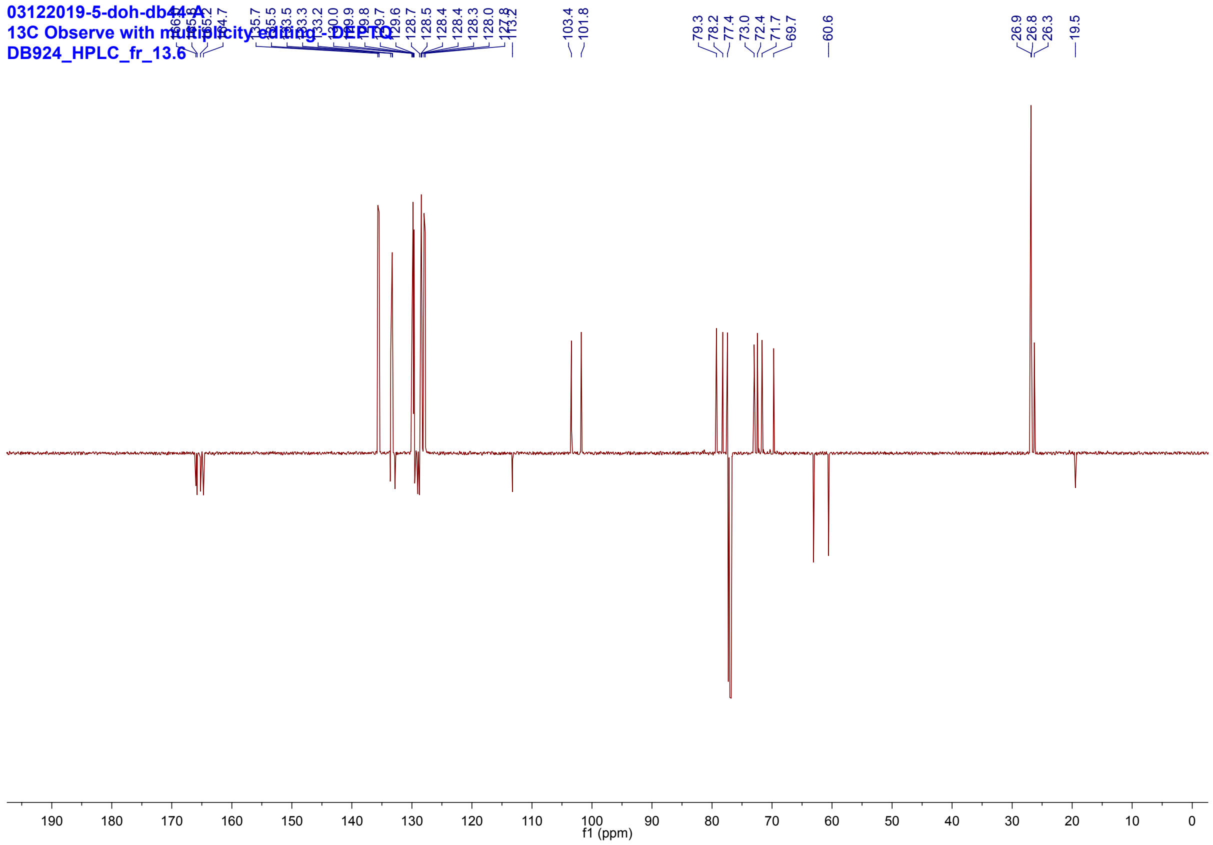

Figure S20. ^{13}C NMR of compound **15**

5-*O*-Tert-butyldiphenylsilyl-3-*O*-(*β*-D-2',3',4',6'-tetra-*O*-benzoylglucopyranosyl)-1,2-*O*-diacetyl-α-D-ribo-furanoside 16

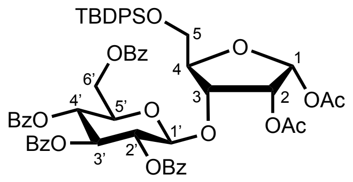

5-*O*-Tert-butyldiphenylsilyl-3-*O*-(*β*-D-2',3',4',6'-tetra-*O*-benzoylglucopyranosyl)-1,2-*O*-isopropylidene-anosribo-furanoside **15** (2.2 g, 2.18 mmol, 1.0 equiv) was dissolved in a 80/20 mixture of acetic acid and water (30 mL) and the mixture heated to reflux (90 ºC) for 4 hours. After cooling to room temperature, the mixture was concentrated under reduced pressure, the residue taken up in ethyl acetate (60 mL) and the organic layer washed with a saturated aqueous solution of sodium hydrogen carbonate (30 mL), saturated brine (30 mL), dried onto magnesium sulfate, filtered and concentrated under reduced pressure to afford a solid residue. This material was dissolved in dry

pyridine (25 mL) under an atmosphere of argon, and acetic anhydride (1.0 mL, 11.0 mmol, 5.0 equiv) was added, and the resulting mixture stirred at room temperature for 16 hours. The mixture was then concentrated under reduced pressure, the residue re-dissolved in ethyl acetate (60 mL) and the organic layer washed with saturated brine (2 x 30 mL), dried onto magnesium sulfate, filtered and concentrated to afford a solid residue. Purification by silica flash column chromatography, eluting with a 20/80 mixture of ethyl acetate and hexane, afforded the title compound as a colourless solid (1.0 g, 46% yield). A very small amount of a more polar compound was also obtained, which was assigned to the α-anomer of the desired compound by NMR analysis. A very pure sample of the title compound was obtained by preparative HPLC purification using a Phenomenex C18 Luna 250 x 21.2 mm column with TMS endcapping, eluting with a 97/3 mixture of acetonitrile and methanol, flow 10 mL/min, 254 nm; Rt = 12.7 min; mp: 61-63 ºC; ^{1}H NMR (500 MHz, $CDCl_3$) δ = 8.12 – 8.05 (m, 2H), 7.96 – 7.90 (m, 2H), 7.86 (ddd, *J*=15.0, 8.3, 1.4, 4H), 7.64 – 7.59 (m, 4H), 7.59 – 7.36 (m, 14H), 7.35 – 7.29 (m, 4H), 6.04 (br s, 1H, *H-2*), 5.86 (t, *J*=9.7, 1H, *H-3'*), 5.69 (t, *J*=9.7, 1H, *H-4'*), 5.55 (dd, *J*=9.9, 7.9, 1H, H-2'), 5.38 (d, *J*=4.8, 1H, *H-1*), 4.87 (d, *J*=7.9, 1H, *H-1'*), 4.70 – 4.61 (m, 2H, *H-3* + *H-6'a*), 4.45 (dd, *J*=12.2, 4.9, 1H, *H-6'b*), 4.11- 4.06 (m, 2H, *H-4* + *H-5'*), 3.81 (dd, *J*=11.8, 2.8, 1H, *H-5a*), 3.57 (dd, *J*=11.8, 3.0, 1H, *H-5b*), 2.07 (s, 3H, *OAc-1*), 1.80 (s, 3H, *OAc-2*), 1.06 (s, 9H, *t*-Bu); ^{13}C NMR (126 MHz, $CDCl_3$) δ = 169.8, 169.4, 166.2, 165.9, 165.2, 165.0, 135.6, 135.6, 133.6, 133.5, 133.4, 133.3, 133.1, 133.1, 130.2, 130.0, 129.9, 129.88, 129.8, 129.7, 129.01, 128.8, 128.6, 128.57, 128.5, 128.47, 128.1, 127.9, 101.5, 98.0, 82.5, 77.4, 75.7, 75.1, 72.8, 72.4, 71.83, 69.5, 63.0, 62.2, 27.0, 21.0, 20.8, 19.5. *m/z* (ESI$^+$): Found $[M+Na]^+$ 1073.3371. $C_{59}H_{58}NaO_{16}Si$ requires M^+, 1073.3386.

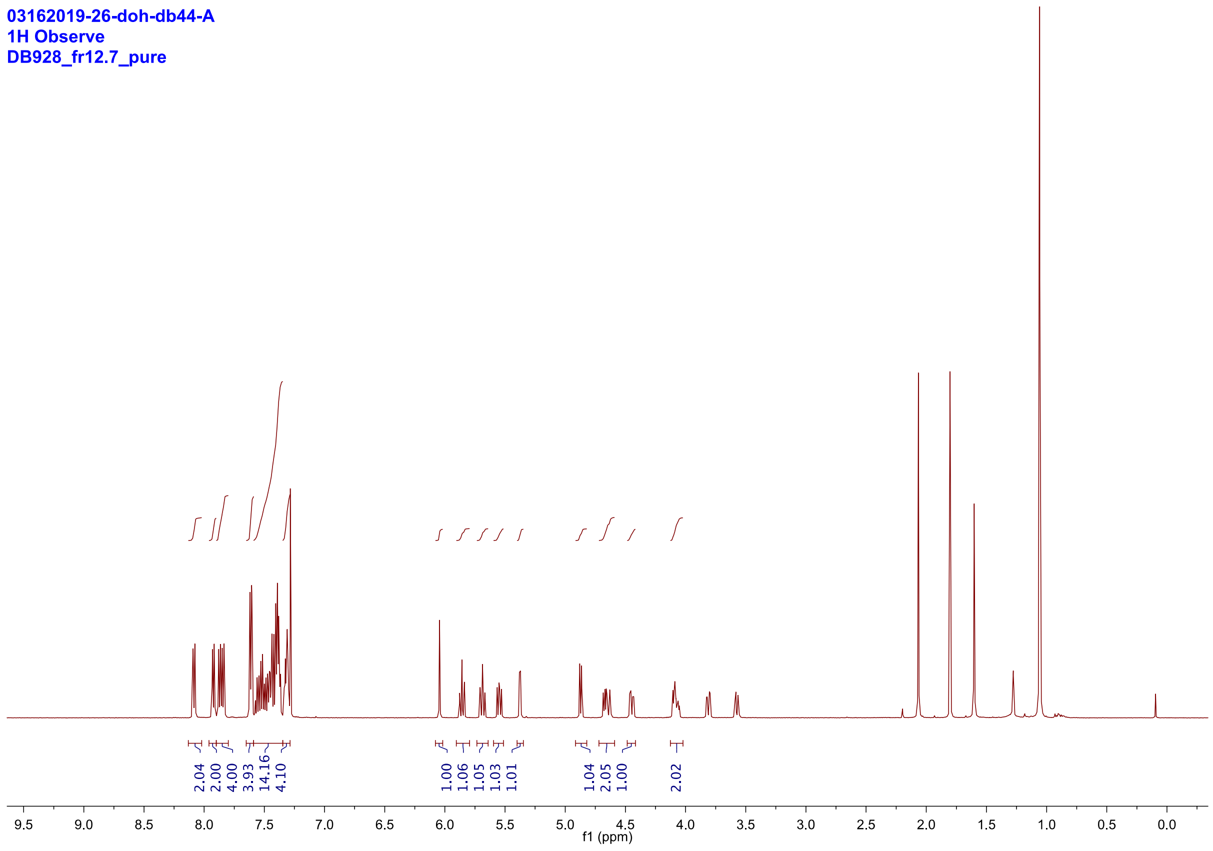

Figure S21. ^{1}H NMR of compound **16**

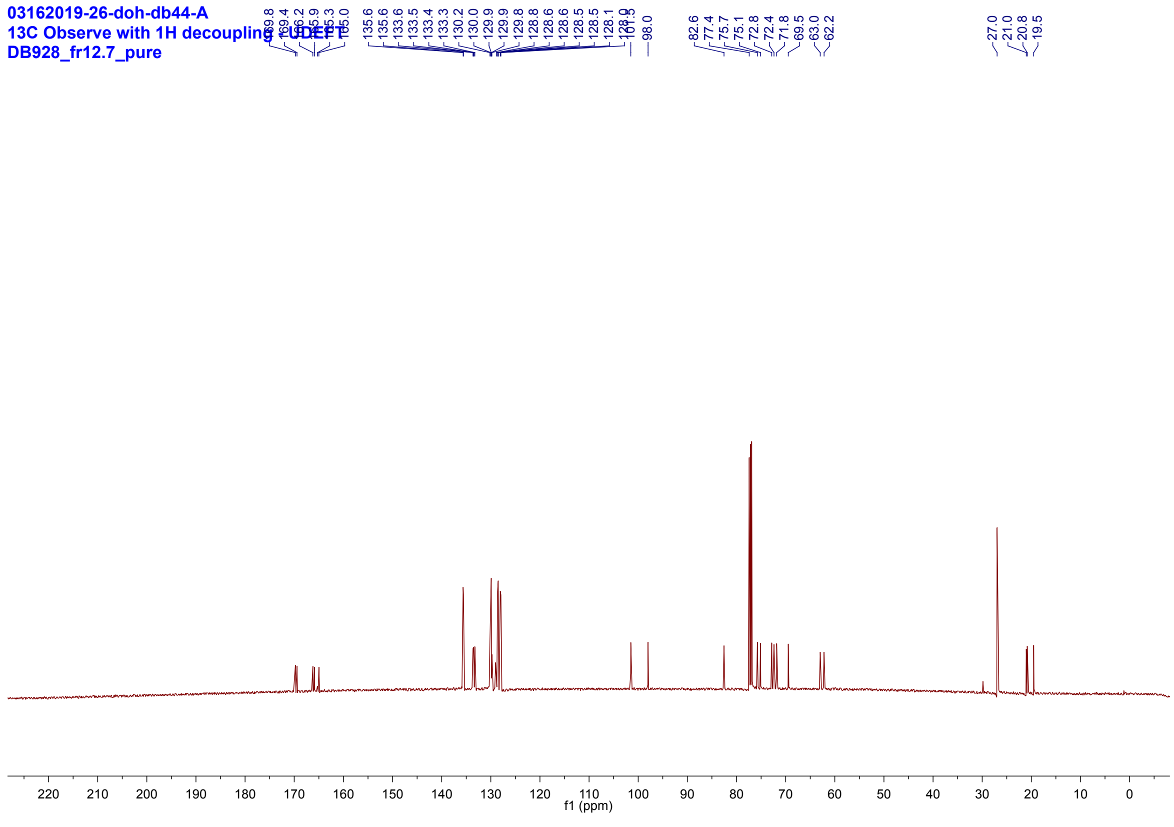

Figure S22. ^{13}C NMR of compound **16**

2'-Acetyl-5'-*O*-tert-butyldiphenylsilyl-3'-*O*-(*β*-D-2'',3'',4'',6''-tetra-*O*-benzoylglucopyranosyl)-6-chloro-9-*β*-D-ribo-furanosylpurine 17

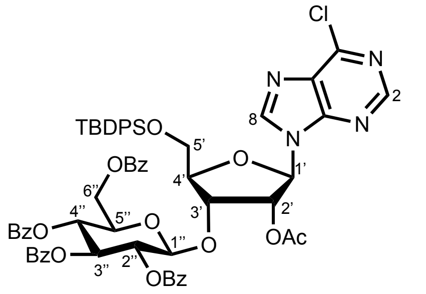

5-*O*-Tert-butyldiphenylsilyl-3-*O*-(β-D-2',3',4',6'-tetra-*O*-benzoylglucopyranosyl)-1,2-*O*-diacetyl-ucopribo-furanoside **16** (740 mg, 0.7 mmol, 1.0 equiv) was dissolved in acetonitrile (55 mL) under an atmosphere of argon. 6-Chloropurine (218 mg, 1.41 mmol, 2.0 equiv) was added, followed by *N,O*-bis(trimethylsilyl)acetamide (860 µL, 3.52 mmol, 5.0 equiv), and the resulting mixture was heated to reflux (80 ºC) for 2 hours. The mixture was then cooled to room temperature, and trimethylsilyl trifluoromethanesulfonate (281 µL, 1.55 mmol, 2.2 equiv) was added. The mixture was then heated to 70 ºC for 16 hours, after which time it was cooled to room temperature and concentrated under reduced pressure. The residue was taken up in ethyl acetate (50 mL), the organic layer washed with a saturated aqueous solution of sodium hydrogen carbonate (30 mL), saturated brine (30 mL), dried onto magnesium sulfate, filtered and concentrated to afford a solid residue. Purification by silica flash chromatography, eluting with a 20/80 mixture of ethyl acetate and hexane afforded the desired compound as a colourless solid (280 mg, 35% yield). A very pure sample was obtained by preparative HPLC purification using a Phenomenex C18 Luna 250 x 21.2 mm column with TMS endcapping, eluting with a 97/3 mixture of acetonitrile and methanol, flow 10 mL/min, 254 nm; Rt = 13.3 min; mp = 107-109 ºC ^{1}H NMR (500 MHz, $CDCl_3$) δ = 8.48 (s, 1H, *H-2*), 8.19 (s, 1H, *H-8*), 7.96 – 7.87 (m, 6H), 7.86 – 7.81 (m, 2H), 7.55 – 7.25 (m, 22H), 6.05 (d, *J*=4.6, 1H, *H-1'*), 5.91 – 5.85 (m, 2H*, H-2'* + *H3''*), 5.65 (t, *J*=9.8, 1H, *H-4''*), 5.57 (dd, *J*=9.8, 7.9, 1H, *H-2''*), 4.95 (t, *J*=5.4, 1H, *H-3'*), 4.85 (d, *J*=7.9, 1H, *H-1''*), 4.60 (dd, *J*=12.2, 2.9, 1H, *H-6'a*), 4.39 (dd, *J*=12.3, 5.6, 1H, *H-6'b*), 4.15 – 4.09 (m, 2H, *H-5''* + *H-4'*), 3.86 (dd, *J*=11.8, 3.6, 1H, *H-5'a*), 3.62 (dd, *J*=11.7, 3.5, 1H, *H-5'b*), 2.07 (s, 3H, *OAc-2'*), 0.98 (s, 9H, *t*-Bu);^{13}C NMR (126 MHz, $CDCl_3$) δ = 169.9, 166.1, 165.8, 165.3, 165.1, 152.1, 151.4, 151.1, 144.3, 135.6, 135.5, 133.7, 133.6, 133.5, 133.2, 132.7, 132.6, 132.3, 130.2, 130.1, 130.0, 129.8, 129.8, 129.7, 129.3, 128.9, 128.8, 128.7, 128.6, 128.6, 128.5, 128.3, 128.0, 127.9,

101.8, 87.2, 83.7, 76.9, 74.0, 72.8, 72.7, 71.8, 69.5, 63.0, 62.5, 27.0, 20.8, 19.3; *m/z* (ESI$^+$): Found [M+Na]$^+$ 1167.3192. $C_{62}H_{57}ClN_4NaO_{14}Si$ requires M^+, 1167.3221.

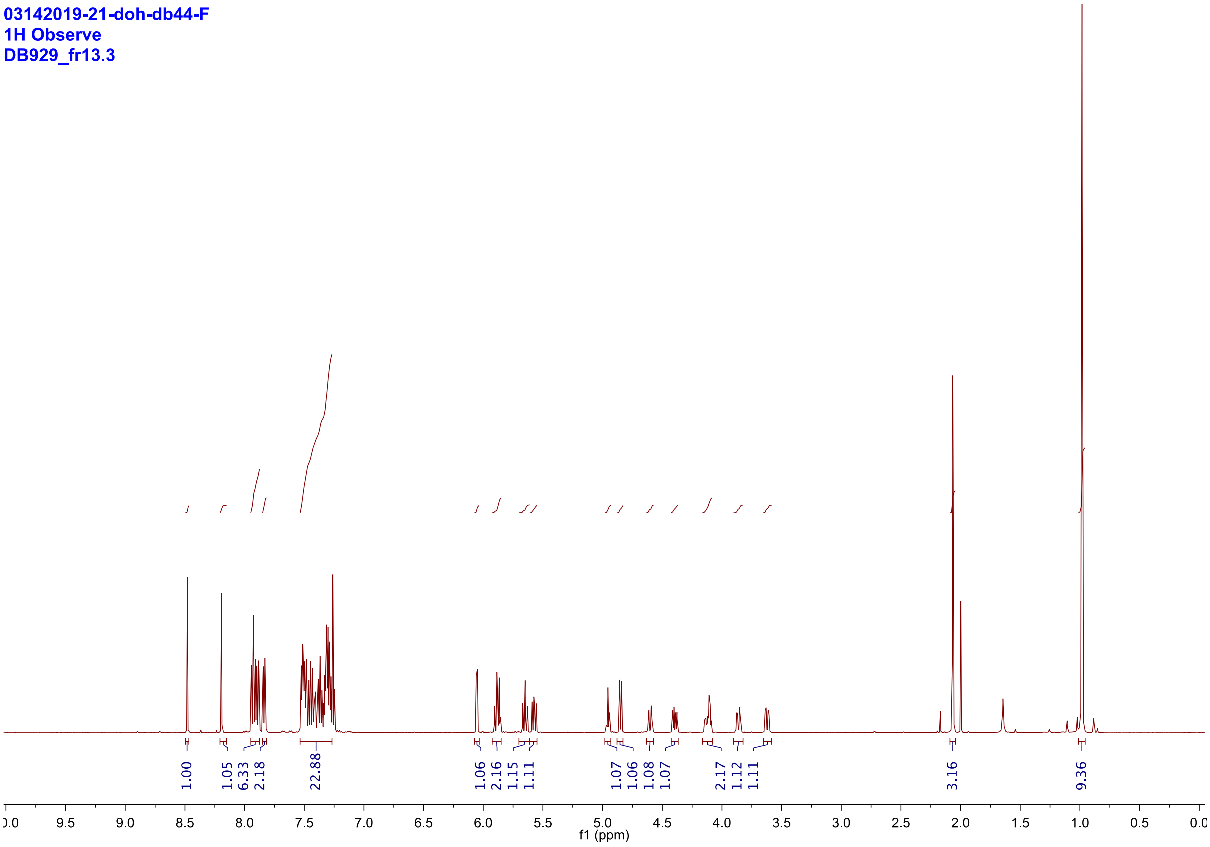

Figure S23. ^{1}H NMR of compound **17**

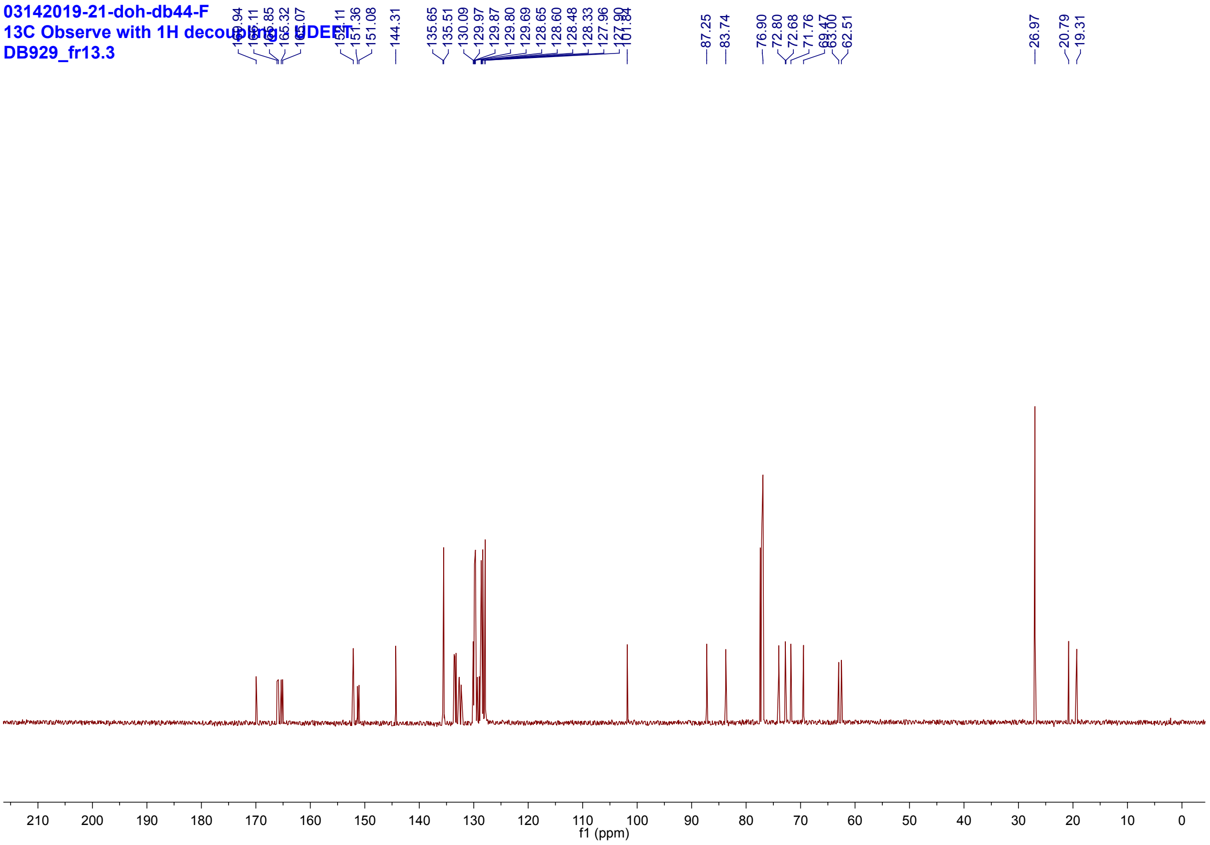

Figure S24. ^{13}C NMR of compound **17**

2'-Acetyl-3'-*O*-(*β*-D-2'',3'',4'',6''-tetra-*O*-benzoylglucopyranosyl)-6-chloro-9-*β*-D-ribo-furanosylpurine 18

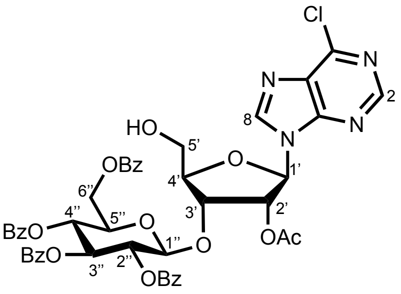

2'-Acetyl-5'-*O*-tert-butyldiphenylsilyl-3'-*O*-(*β*-D-2'',3'',4'',6''-tetra-*O* benzoylglucopyranosyl)-6-chloro-9-*β*-D-ribo-furanosylpurine **17** (70 mg, 0.06 mmol, 1.0 equiv) was dissolved in dry tetrahydrofuran (5 mL) under an atmosphere of argon. The mixture was cooled to 0 ºC and tetrabutylammonium fluoride (1.0 M solution in tetrahydrofuran, 73 μL, 0.073 mmol, 1.2 equiv) was added. The mixture was stirred for 1 hour at 0 ºC, then it was warmed to room temperature and concentrated under reduced pressure. The residue was re-dissolved in ethyl acetate (20 mL and washed with saturated brine (3 x 10 mL), dried onto magnesium sulfate, filtered and

concentrated to afford a solid residue. Purification by silica flash chromatography, eluting with a 20/80 to 30/70 mixture of ethyl acetate and hexane, afforded the desired compound as a colourless solid (36.3 mg, 61% yield). Also, two more compounds were isolated, one being more polar with respect to the title compound and identified by NMR and low-res MS as the deacetylated intermediate **A**, 10 mg, 19% yield, *m/z* (ESI^+) 887 $(M+Na)^+$, (ESI^-) 899 $(M+Cl)^-$, 863 $(M-H)^-$, and the other being less polar with respect to the title compound, and identified by NMR and low-res MS as the diacetyl compound **B**, 9.0 mg, 15% yield, *m/z* (ESI^+) 971 $(M+Na)^+$, 795 $[M-(6\text{-Cl-purine}]^+$, ($ESI^-$) 983 $(M+Cl)^-$.

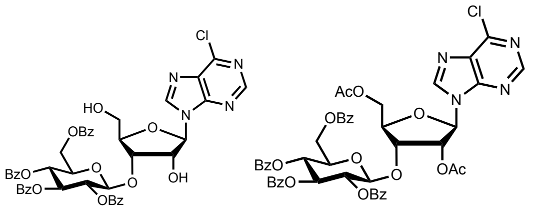

A B

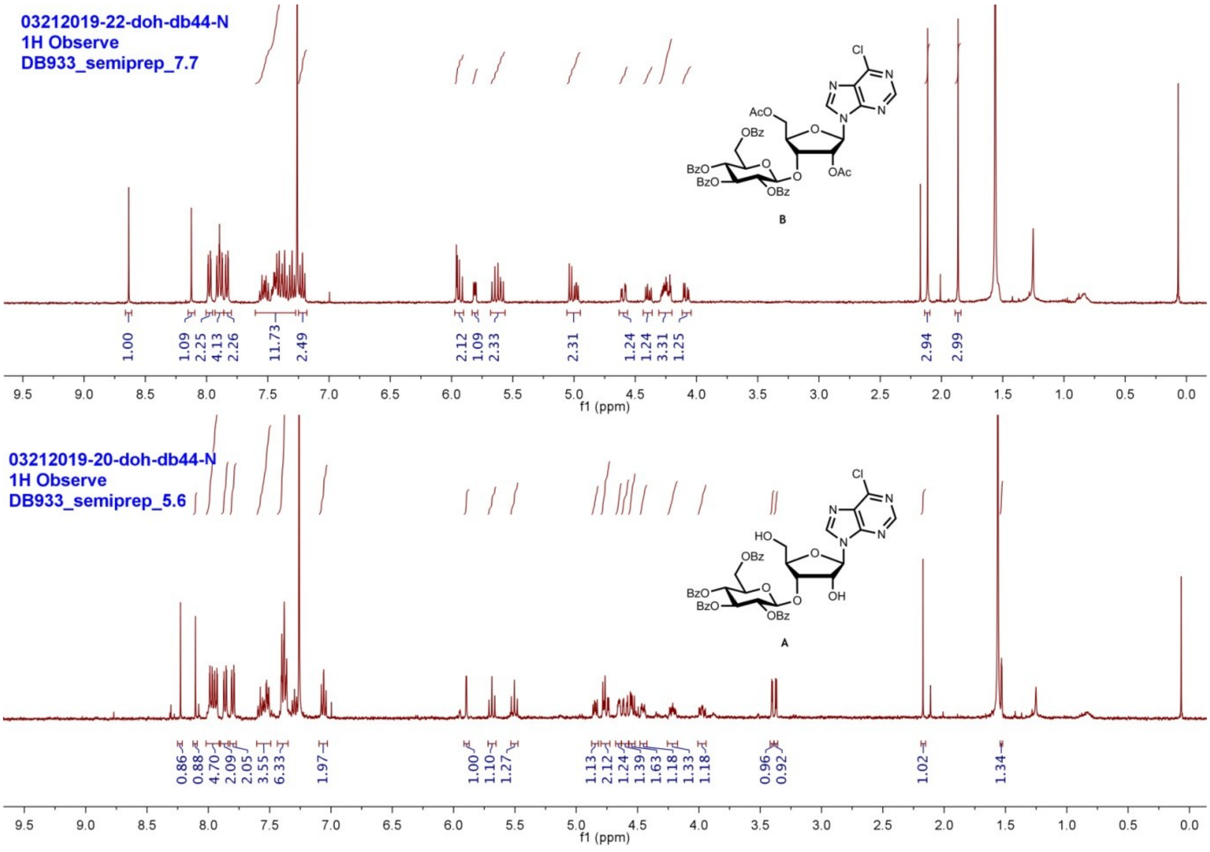

Figure S25. ^{1}H NMR of compounds **18A** and **18B**

A very pure sample of the title compound was obtained by semi-preparative HPLC purification using a Phenomenex C18 Luna 250 x 10 mm column, eluting with an 80/20 mixture of acetonitrile and water, flow 2.5 mL/min, 254 nm; Rt = 6.6 min; mp = 123-125 ºC; ^{1}H NMR (500 MHz, $CDCl_3$) δ = 8.73 (s, 1H, *H-2*), 8.17 (s, 1H, *H-8*), 8.04 – 7.98 (m, 4H), 7.94 – 7.88 (m, 2H), 7.88 – 7.84 (m, 2H), 7.60 – 7.31 (m, 12H), 6.04 (d, *J*=6.4, 1H, *H-1'*), 5.93 (t, *J*=9.7, 1H, *H-3''*), 5.74 (t, *J*=9.7, 1H, *H-4''*), 5.68 (dd, *J*=6.4, 5.5, 1H, *H-2'*), 5.63 (dd, *J*=9.8, 7.8, 1H, *H-2''*), 5.01 (d, *J*=7.8, 1H, *H-1''*), 4.94 (dd, *J*=5.5, 3.0, 1H, *H-3'*), 4.86 – 4.78 (m, 1H, *OH-5'*), 4.62 (dd, *J*=12.4, 2.9, 1H, *H-6''a*), 4.49 (dd, *J*=12.3, 5.0, 1H, *H-6''b*), 4.22 – 4.16 (m, 2H, *H-5'' + H-4'*), 3.88 – 3.81 (m, 1H, *H-5'a*), 3.63 – 3.55 (m, 1H, *H-5'b*), 2.03 (s, 3H, *OAc-2'*); ^{13}C NMR (126 MHz, $CDCl_3$) δ = 169.8, 166.0, 165.7, 165.1, 165.1, 152.4, 151.6, 150.4, 144.9, 133.7, 133.6, 133.4, 133.3, 129.8, 129.8, 129.7, 129.7, 129.2, 128.8, 128.6, 128.6, 128.5, 128.4, 101.9, 88.9, 86.1, 78.2, 73.9, 72.7, 71.9, 69.1, 62.7, 62.1, 20.5; *m/z* (ESI$^+$): Found [M+Na]$^+$ 929.2018. $C_{46}H_{39}ClN_4NaO_{14}$ requires M^+, 929.2044.

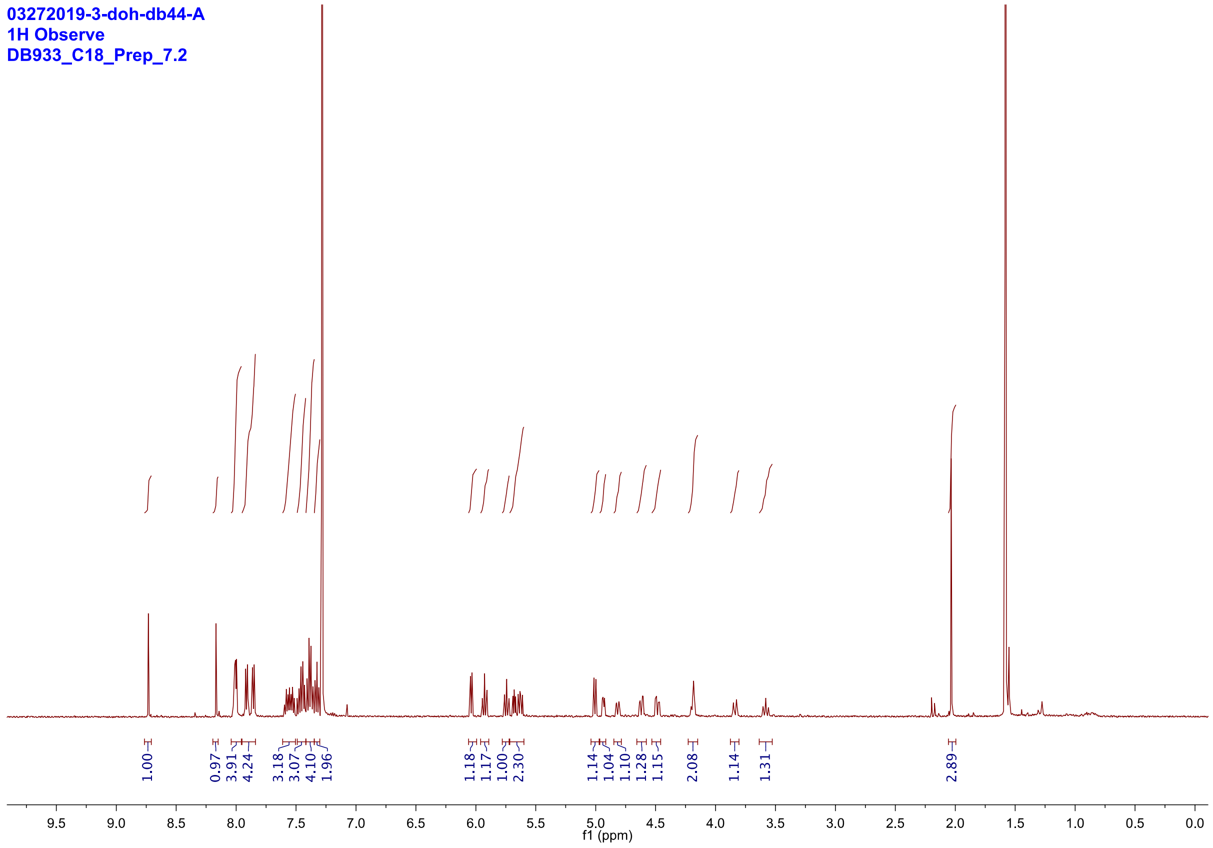

Figure S26. ^{1}H NMR of compound **18**

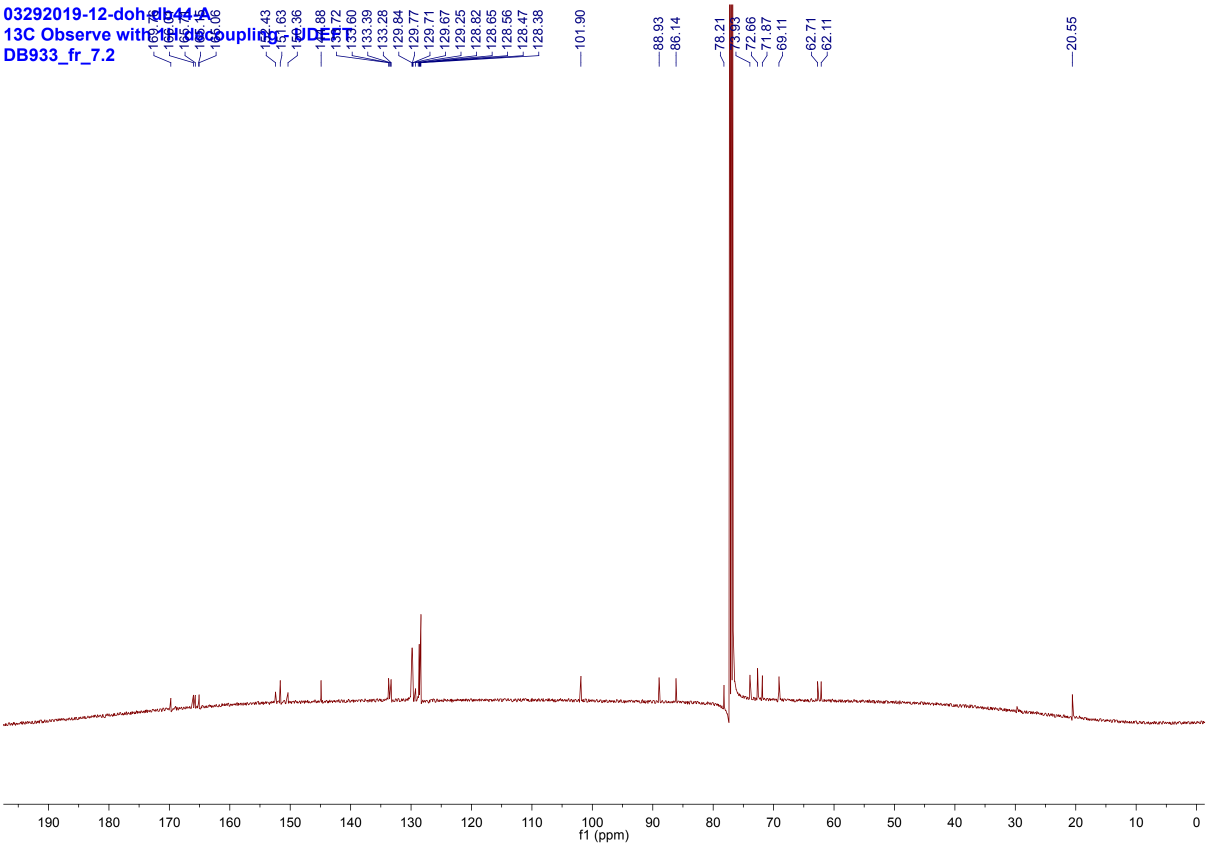

Figure S27. ^{13}C NMR of compound **18**

3′ -*O*-(*β*-D-Glucopyranosyl)-adenosine 11

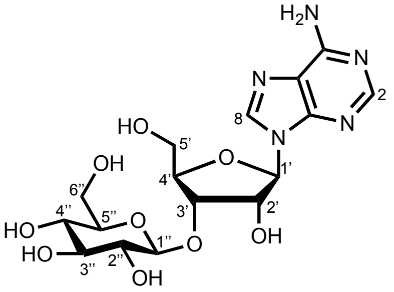

The final synthetic step involved global removal of the protecting groups. Therefore, in order to increase the final yields, along with compound 2’-acetyl-3’-*O*-(*β*-D-2’’,3’’,4’’,6’’-tetra-*O*-benzoylglucopyranosyl)-6-chloro-9-*β*-D-ribo-furanosylpurine **18**, compounds **A** and **B** were also used.

Thus, a mixture of compounds **18** (21.0 mg, 0.023 mmol, 0.52 equiv), **A** (10.0 mg, 0.012 mmol, 0.27 equiv) and **B** (9.0 mg, 0.009 mmol, 0.21 equiv) were loaded in a heavy walled pressure flask and in ice cold ethanol (5 mL) which had previously been saturated with ammonia by bubbling the gas for 30 min at 0 ºC, was added. The flask was sealed and the mixture was heated at 80 ºC for 24h. The mixture was then cooled

to room temperature, the solvent removed under reduced pressure and the residue purified semi-preparative HPLC purification using a Phenomenex C18 Luna 250 x 10 mm column, eluting with an 10/90 mixture of acetonitrile and water, flow 2.5 mL/min, 260 nm, furnishing after freeze drying the title compound as a colourless foam; Rt = 8.3 min. ^{1}H NMR (500 MHz, D_2O) δ = 8.48 (s, 1H, *H-2*), 8.38 (s, 1H, *H-8*), 6.16 (d, *J*=4.9, 1H, *H-1'*), 4.87 (t, *J*=5.1, 1H, *H-2'*), 4.61 (t, *J*=4.9, 1H, *H-3'*), 4.59 (d, *J*=7.8, 1H, *H-1''*), 4-41 - 4.39 (m, 1H, *H-4'*), 3.92 (dd, *J*=12.8, 3.1, 1H, *H-5'a*), 3.88 – 3.86 (m, 1H, *H-6''a*), 3.83 (dd, *J*=13.03, 4.03, 1H, *H-5'b*), 3.71 (dd, *J*=12.4, 5.0, 1H, *H-6''b)*, 3.50 (t, *J*=8.89*,* 1H, *H-4''*), 3.45 – 3.36 (m, 3H, *H-5''* + *H-3''* + *H-2''*); ^{13}C NMR (126 MHz, D_2O) δ = 151.26, 148.17 (*C-8*), 146.28, 142.22 (*C-2*), 118.98, 101.95 (*C-1''*), 88.60 (*C-1'*), 83.66 (*C-4'*), 77.51 (*C-3'*), 75.85 (*C-5''*), 75.40 (*C-4''*), 73.50 (*C-2'*), 72.67 *(C-2''*), 69.33 *(C-3''*), 60.79 (*C-5'*), 60.41 *(C-6'')*; *m/z* (ESI$^+$): Found [M+H]$^+$ 430.1562. $C_{16}H_{24}N_5O_9$ requires M^+, 430.1569.

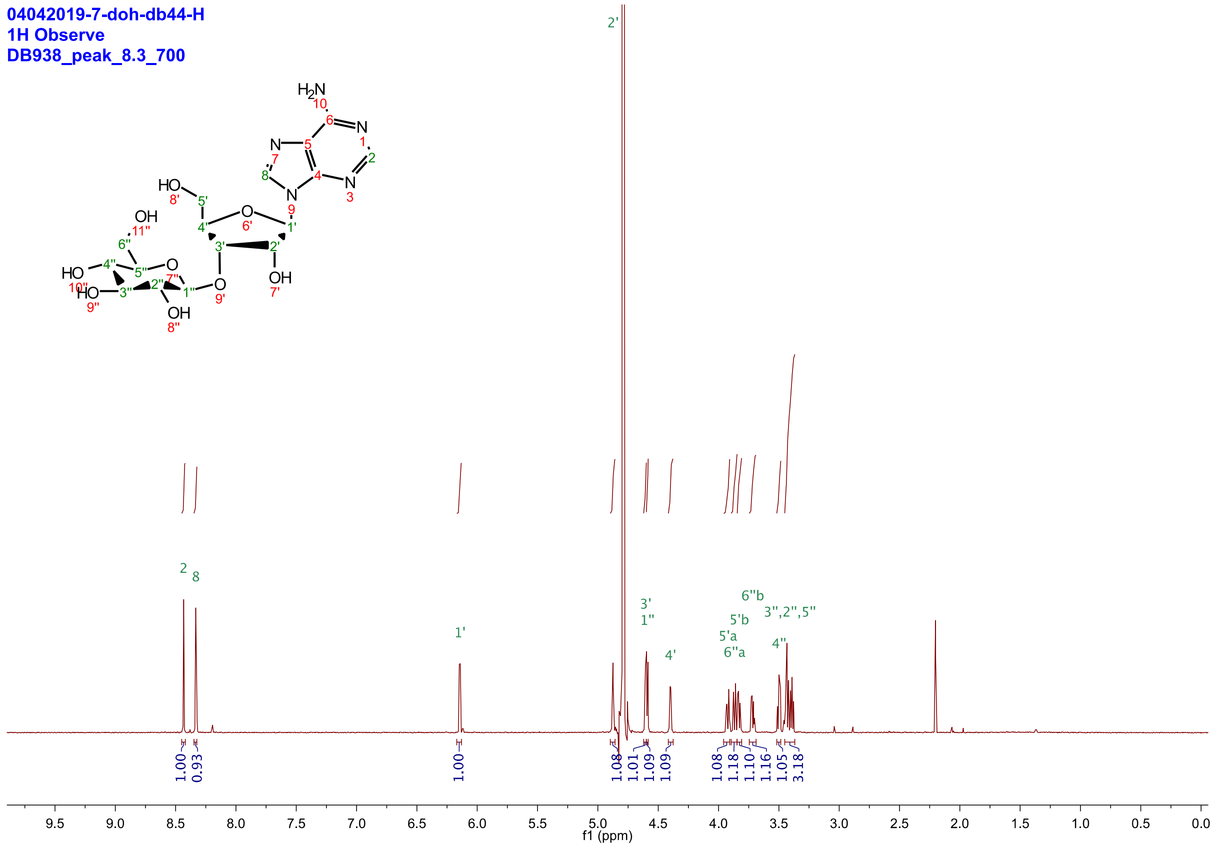

Figure S28. ^{1}H NMR of compound **11**

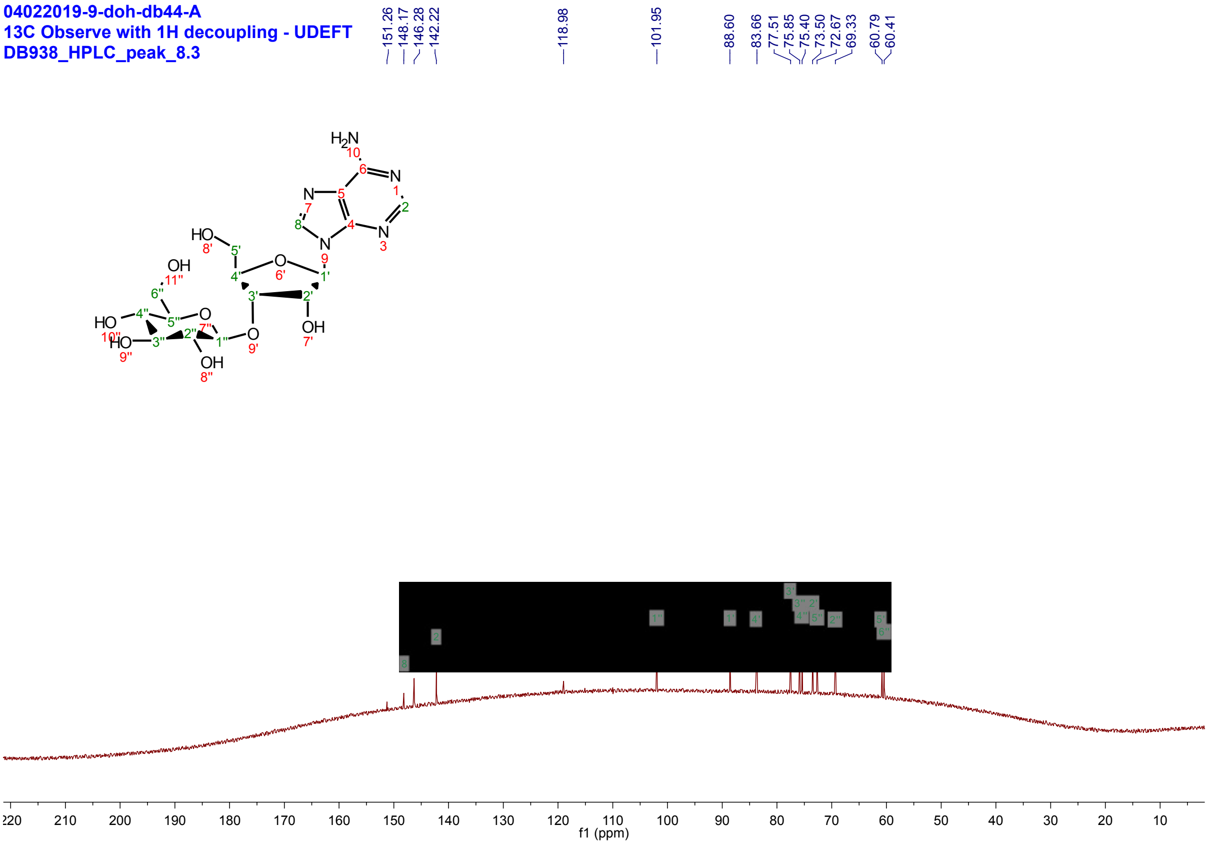

Figure S29. ^{13}C NMR of compound **11**

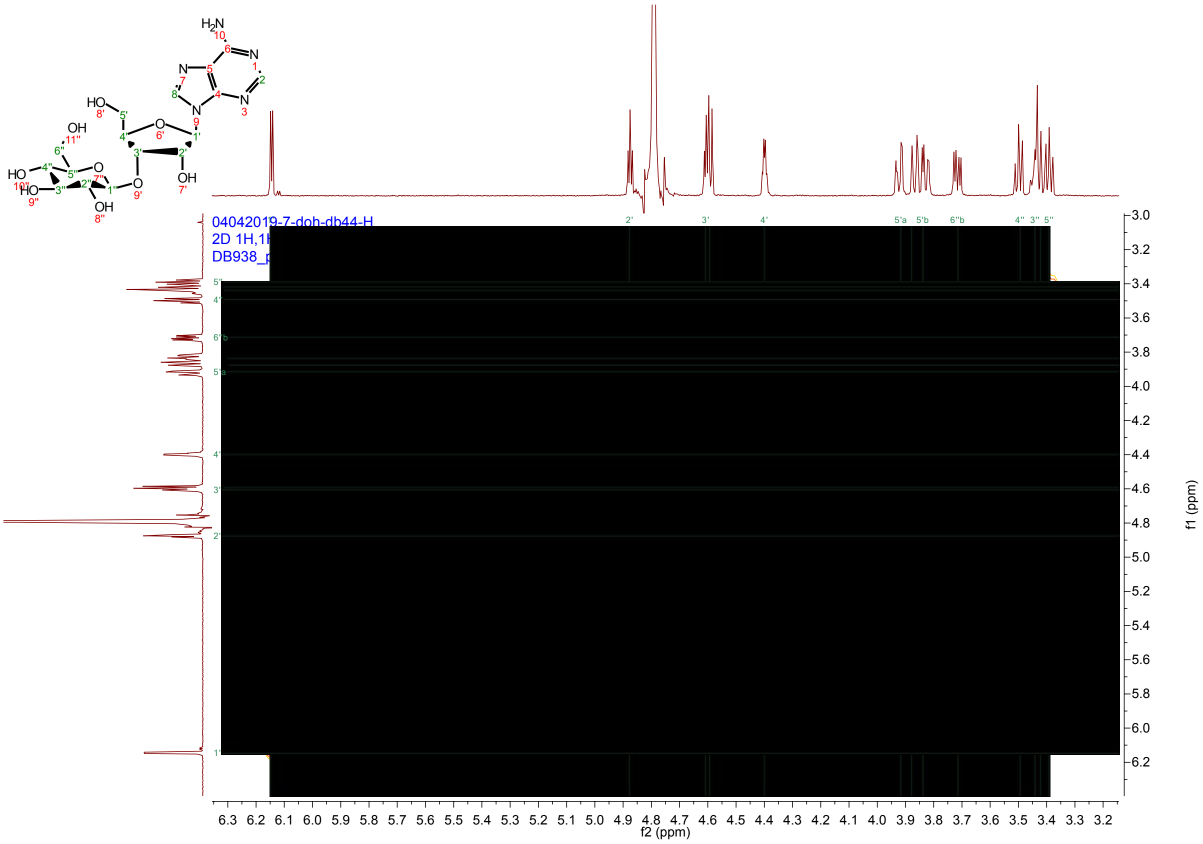

Figure S30. ^{1}H-^{1}H COSY of compound **11**.

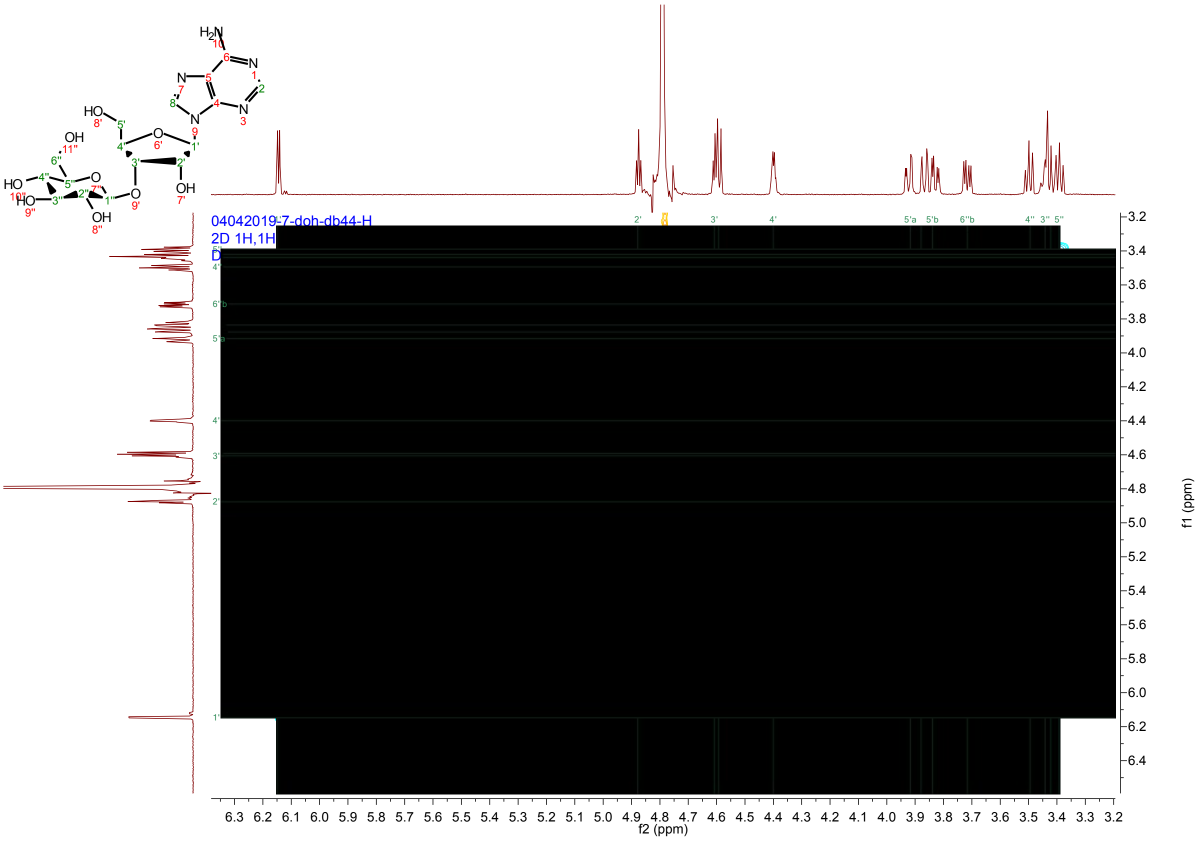

Figure S31. ^{1}H-^{1}H TOCSY of compound **11**.

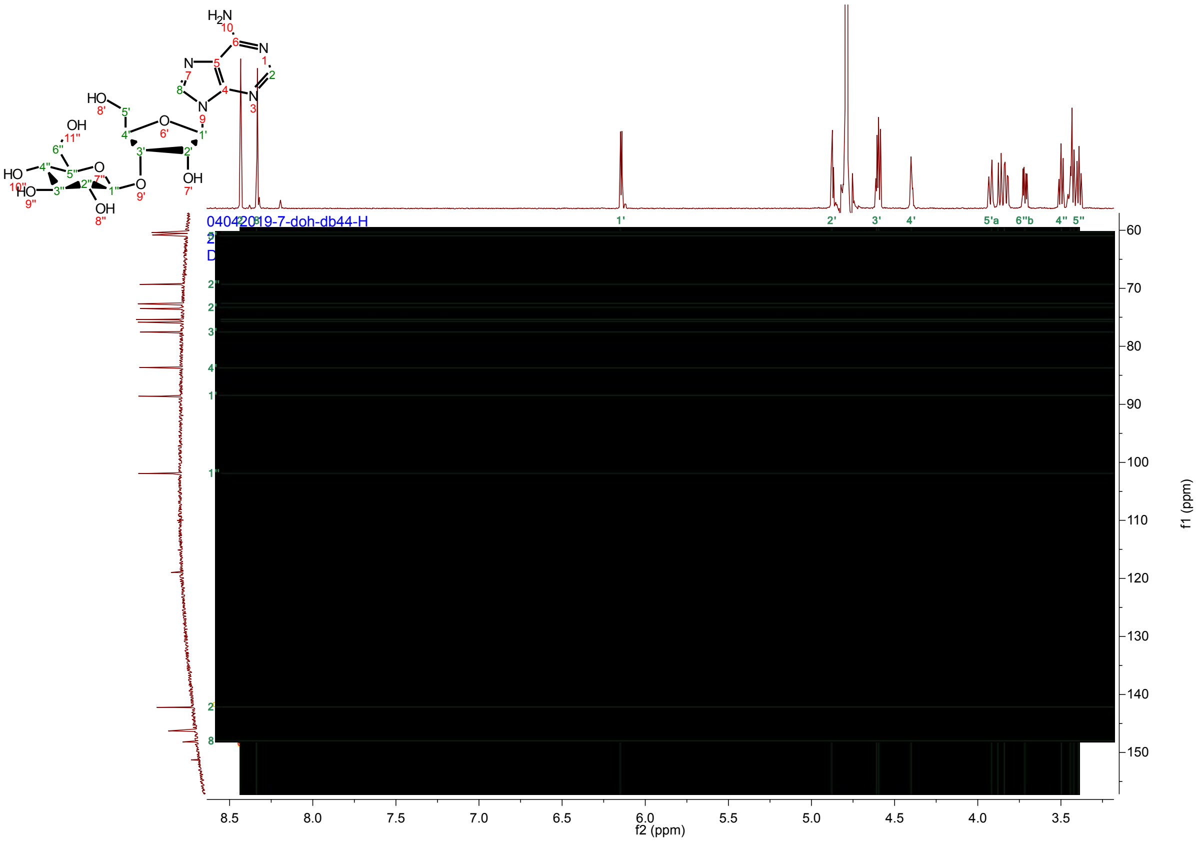

Figure S32. ^{1}H-^{13}C HMBC of compound **11**.

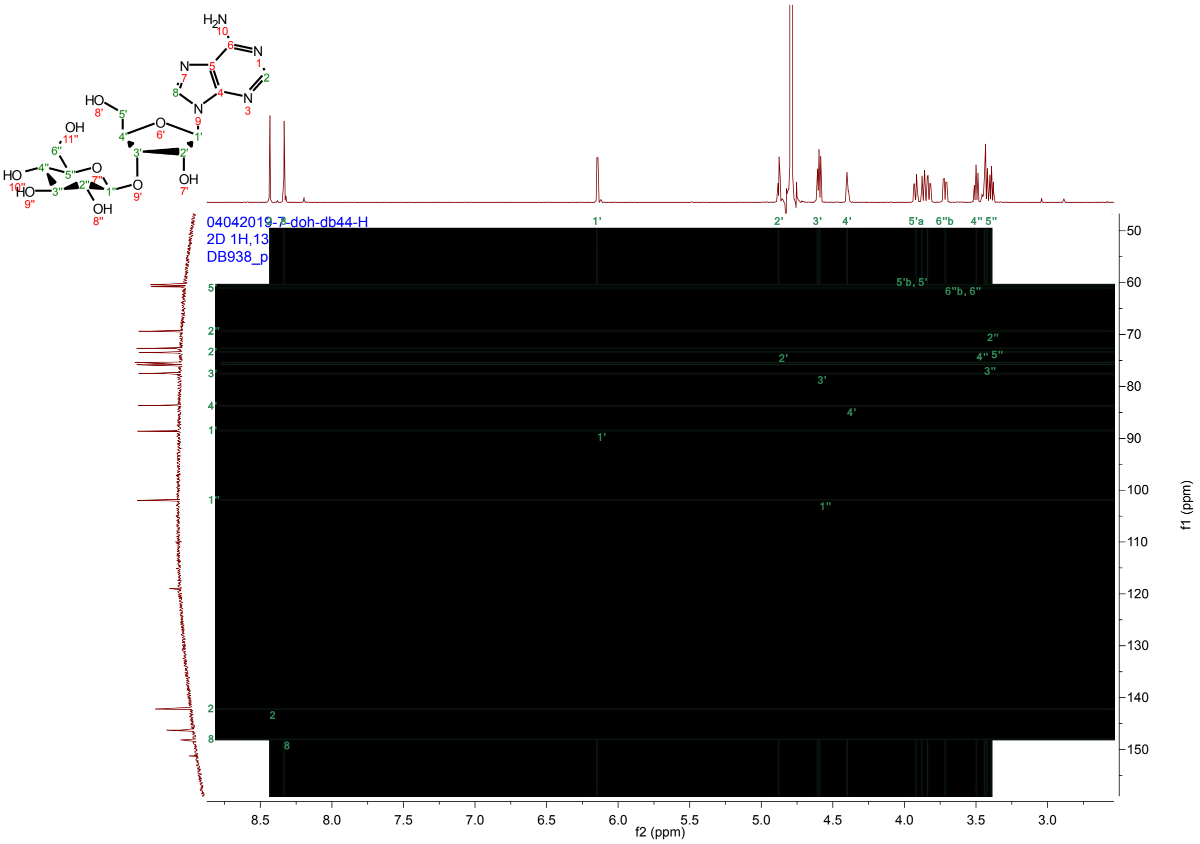

Figure S33. ^{1}H-^{13}C HSQC of compound **11**.

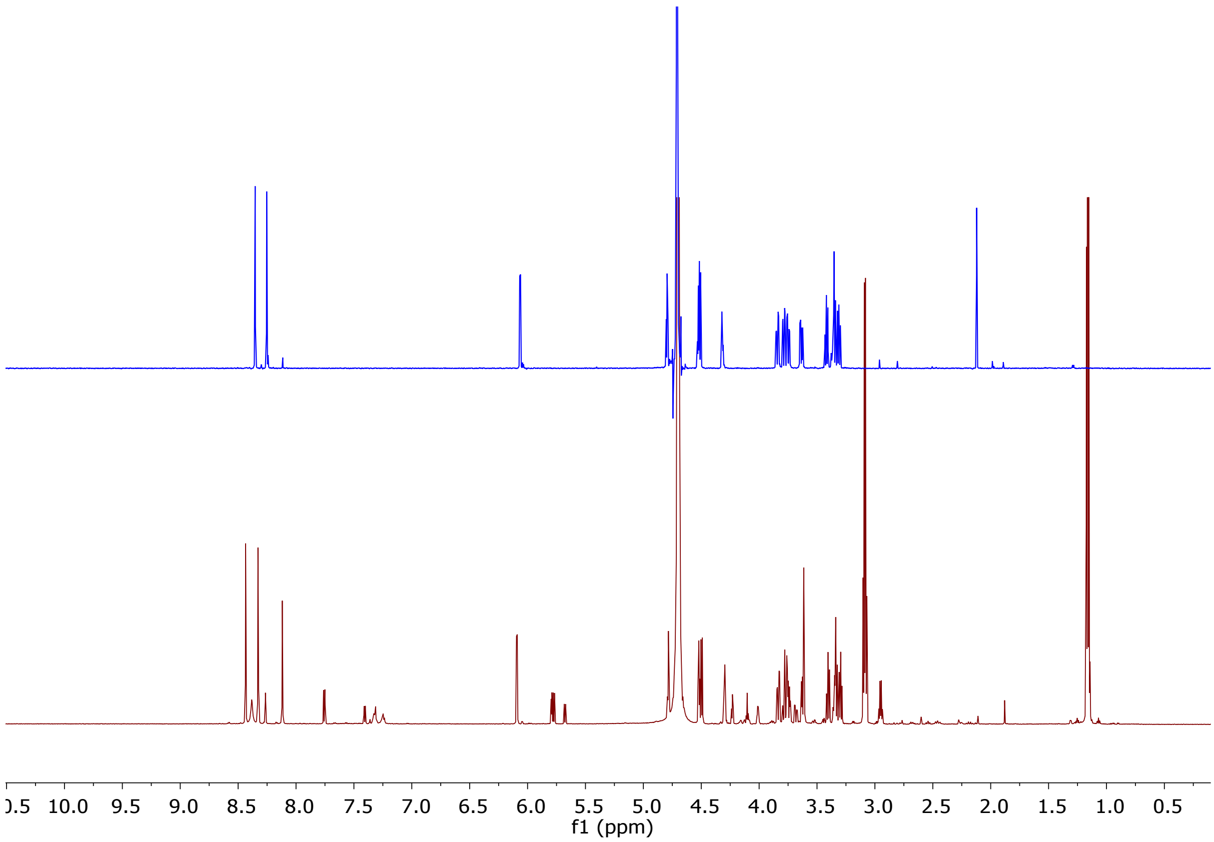

Figure S34. Stacked ^{1}H NMR of chemically (Top - Blue) and enzymatically (Bottom - Red) synthesised compound **11**.

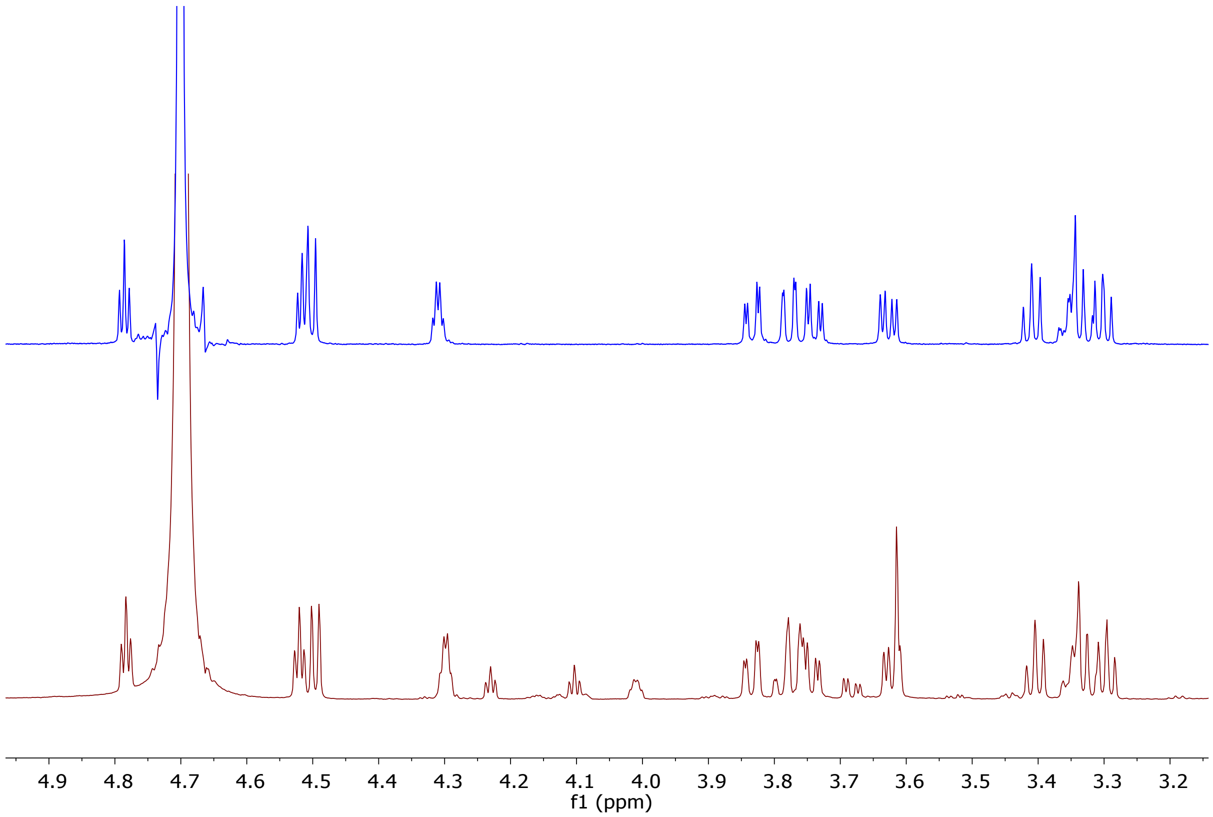

Figure S35. Zoomed - Stacked ^{1}H NMR of chemically (Top - Blue) and enzymatically (Bottom - Red) synthesised compound **11**

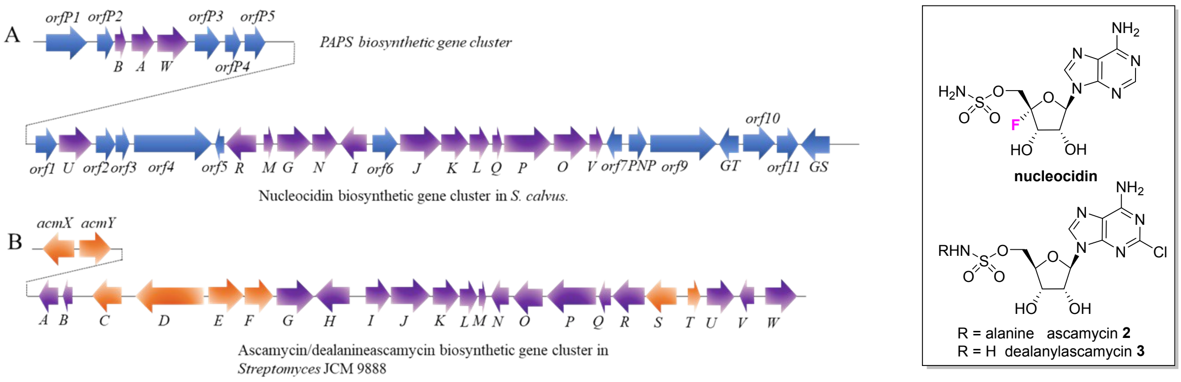

.

orfP1	565	Ferredoxin-sulfite reductase	*nucN*	332	Amidinotransferase
orfP2	236	PAPS reductase	*nucI*	389	Sulfatase
nucB	178	Adenylyl-sulfate kinase	*orf6*	348	StrR-transcriptional regulator
nucA	311	Sulfate adenylyltransferase CysD	*nucJ*	560	Radical SAM/B_{12} superfamily
nucW	444	Sulfate adenylyltransferase subunit	*nucK*	359	Sulfotransferase domain
orfP3	367	Sulfonate ABC transporter	*nucL*	255	SAM-Me-transferase
orfP4	262	ABC transporter ATP-binding protein	*nucQ*	158	Rubrerythrin
orfP5	296	ABC transporter permease	*nucP*	661	SAM-Methyltransferase
orf1	332	Oxidoreductase	*nucO*	460	sulfotransferase
nucU	476	Cation: H^+ antiporter	*nucV*	194	Adenphosphoribosyltransferase
orf2	275	Hypothetical protein	*orf7*	205	LuxR transcription regulator
orf3	194	Histidine phosphatase family protein	*PNP*	273	5'-MTA phosphorylase
orf4	1056	Transcriptional regulatory protein	*orf9*	894	Lycopene cyclase
orf5	137	Aminoglycoside phosphotransferase	*nucGT*	305	Glucosyltransferase
nucR	462	Metabolite transport protein YhjE	*orf10*	612	Hypothetical protein
nucM	140	Hypothetical protein	*orf11*	360	Protein kinase domain- protein
nucG	473	Sulfatase	*nucGS*	479	*β*-glucosidase

Relationship of the biosynthetic gene clusters for **A** nucleocidin[8] and **B** ascamycins.[9] The genes in **purple** are found in both clusters; the **blue** genes only exist for nucleocidin (*S. calvus*) and the **orange** genes only for ascamycin (*Streptomyces JCM 9888*). The numbers refer to size (a.a).

Figure S36. The biosynthetic gene cluster of nucleocidin in *S. calvus* and its comparison with the ascamycin gene cluster.

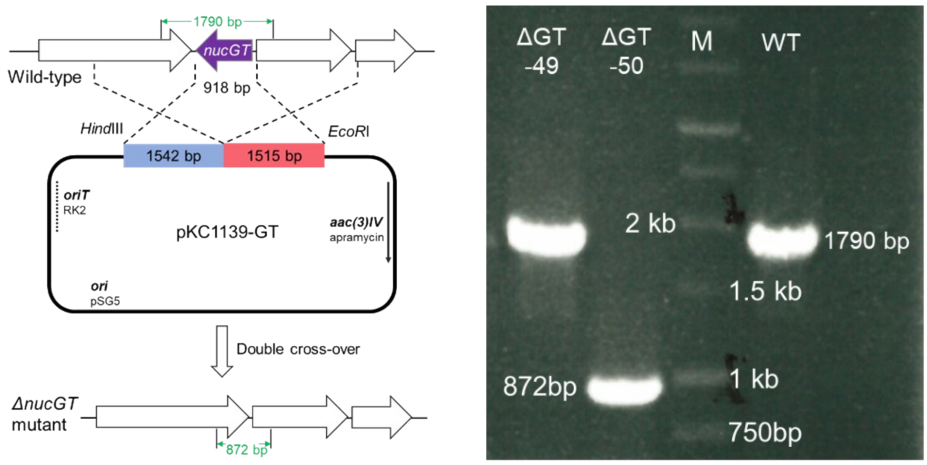

Figure S37. Generation and validation of *nuc-GT* knock-out mutant.

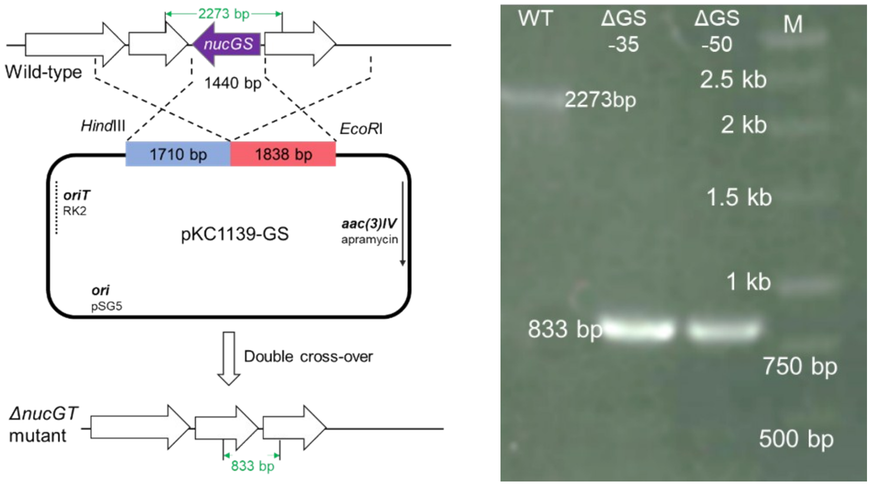

Figure S38. Generation and validation of *nuc-GS* knock-out mutant.

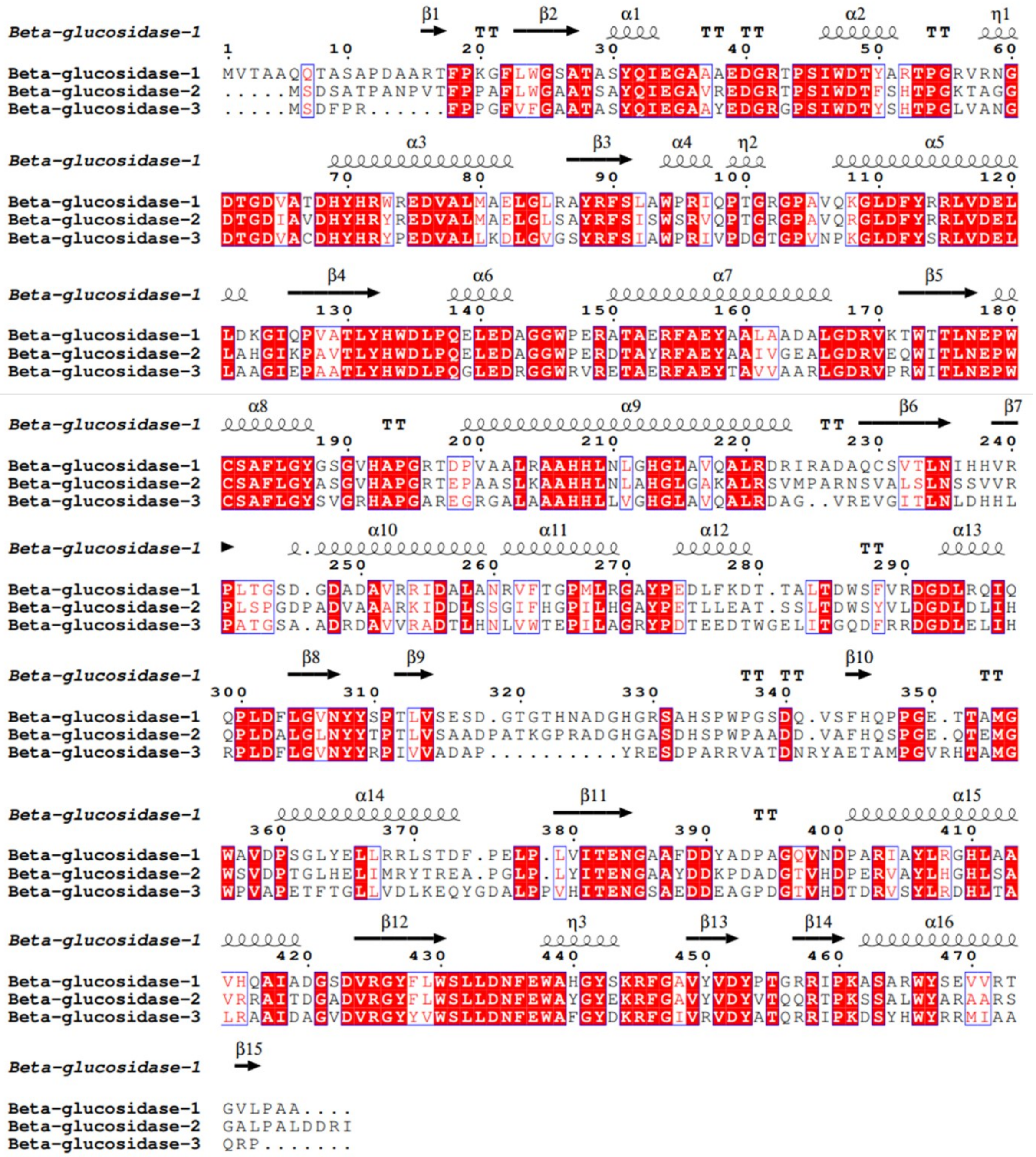

Figure S39. The alignment of *nucGS* and two other β-glucosidases in *S. calvus* genome. PDB 1GNX was used as a template for predicting secondary structures.[7] The average consensus amino acid sequence of these three glycosidases is ~ 88%.

References

1 D. D. Perrins, W. L. F. Armarego, *Purification of Laboratory Chemicals*, Pergamon Press, Oxford, **1988**.

2 P. Mujumdar, S. Bua, C. T. Supuran, T. S. Peat, S-A. Poulsen, *Biorg. Chem. Med. Lett.*, 2018, **28**, 3009 -3013.

3 F. Egami, N. Takahashi, Bull. *Chem. Soc. Jpn.*, 1955, **28**, 666-668.

4 B. J. Beahm, K. W. Dehnert, N. L. Derr, J. Kuhn, J. K. Eberhart, D. Spillmann, S. L. Amacher, and C. R. Bertozzi*; *Angew. Chem. Int. Ed.* 2014, **53**, 3347-3352.

5 W. Pilgrim, P. V. Murphy, *J. Org. Chem.*, 2010, **75**, 6747-6755.

6 T. Murakami, Y. Sato and M. Shibakami, *Carbohydr. Res.*, 2008, **343**, 1297-1308.

7 X. Robert, P. Gouet, *Nucleic Acids Res*, 2014, **42**, 320–324.

8 X. M. Zhu, S. Hackl, M. N. Thaker, L. Kalan, C. Weber, D. S. Urgast, E. M. Krupp, A. Brewer, S. Vanner, A. Szawiola, G. Yim, J. Feldmann, A. Bechthold, G. D. Wright, D. L. Zechel, *ChemBioChem.*, 2015, **16**, 2498 – 2506.

9 C. Zhao, J. Qi, W. Tao, L. He, W. Xu, J. Chen, Z. Deng, *PLOS ONE*, 2014, **9**, e114722.
